# The Mammalian Cell Cycle Regulates Parvovirus Nuclear Capsid Assembly

**DOI:** 10.1371/journal.ppat.1004920

**Published:** 2015-06-11

**Authors:** Jon Gil-Ranedo, Eva Hernando, Laura Riolobos, Carlos Domínguez, Michael Kann, José M. Almendral

**Affiliations:** 1 Centro de Biología Molecular "Severo Ochoa" (Consejo Superior de Investigaciones Científicas-Universidad Autónoma de Madrid), Cantoblanco, Madrid, Spain; 2 University of Bordeaux, Microbiologie Fondamentale et Pathogénicité, UMR 5234, Bordeaux, France; 3 CNRS, Microbiologie Fondamentale et Pathogénicité, UMR 5234, Bordeaux, France; 4 Centre Hospitalier Universitaire de Bordeaux, Service de Virologie, Bordeaux, France; University of North Carolina at Chapel Hill, UNITED STATES

## Abstract

It is unknown whether the mammalian cell cycle could impact the assembly of viruses maturing in the nucleus. We addressed this question using MVM, a reference member of the icosahedral ssDNA nuclear parvoviruses, which requires cell proliferation to infect by mechanisms partly understood. Constitutively expressed MVM capsid subunits (VPs) accumulated in the cytoplasm of mouse and human fibroblasts synchronized at G0, G1, and G1/S transition. Upon arrest release, VPs translocated to the nucleus as cells entered S phase, at efficiencies relying on cell origin and arrest method, and immediately assembled into capsids. In synchronously infected cells, the consecutive virus life cycle steps (gene expression, proteins nuclear translocation, capsid assembly, genome replication and encapsidation) proceeded tightly coupled to cell cycle progression from G0/G1 through S into G2 phase. However, a DNA synthesis stress caused by thymidine irreversibly disrupted virus life cycle, as VPs became increasingly retained in the cytoplasm hours post-stress, forming empty capsids in mouse fibroblasts, thereby impairing encapsidation of the nuclear viral DNA replicative intermediates. Synchronously infected cells subjected to density-arrest signals while traversing early S phase also blocked VPs transport, resulting in a similar misplaced cytoplasmic capsid assembly in mouse fibroblasts. In contrast, thymidine and density arrest signals deregulating virus assembly neither perturbed nuclear translocation of the NS1 protein nor viral genome replication occurring under S/G2 cycle arrest. An underlying mechanism of cell cycle control was identified in the nuclear translocation of phosphorylated VPs trimeric assembly intermediates, which accessed a non-conserved route distinct from the importin α2/β1 and transportin pathways. The exquisite cell cycle-dependence of parvovirus nuclear capsid assembly conforms a novel paradigm of time and functional coupling between cellular and virus life cycles. This junction may determine the characteristic parvovirus tropism for proliferative and cancer cells, and its disturbance could critically contribute to persistence in host tissues.

## Author Summary

Cellular and viral life cycles are connected through multiple, though poorly understood, mechanisms. Parvoviruses infect humans and a broad spectrum of animals, causing a variety of diseases, but they are also used in experimental cancer therapy and serve as vectors for gene therapy. Parvoviruses can only multiply in proliferating cells providing essential replicative and transcriptional functions. However, it is unknown whether the cell cycle regulatory machinery may also control parvovirus assembly. We found that the nuclear translocation of parvovirus MVM capsid subunits (VPs) was highly dependent on physiological cell cycle regulations in mammalian fibroblasts, including: quiescence, progression through G1/S boundary, DNA synthesis, and cell to cell contacts. VPs nuclear translocation was significantly more sensitive to cell cycle controls than viral genome replication and gene expression. The results support nuclear capsid assembly as the major driving process of parvoviruses biological hallmarks, such as pathogenesis in proliferative tissues and tropism for cancer cells. In addition, disturbing the tight coupling of parvovirus assembly with the cell cycle may determine viral persistence in quiescent and post-mitotic host tissues. These findings may contribute to understand cellular regulations on the assembly of other nuclear eukaryotic viruses, and to develop cell cycle-based avenues for antiviral therapy.

## Introduction

Viruses infecting eukaryotes encounter a dynamic regulatory network of proteins expression, posttranslational modifications and signaling, ensuring fine-tuned control of the cell cycle progression and checkpoints [1–3]. These processes include the still poorly understood cell cycle-dependent vast macromolecular traffic across the central aqueous channel of the nuclear pore complex (NPC; [4,5]), which requires soluble receptors (karyopherins) for the recognition of cargos carrying nuclear transport signals [6,7]. All these cell cycle regulations affect the multiplication of many eukaryotic viruses, which must either adapt their life cycles to these factors networks, or perturb them for their benefit. This is particularly important for the many viruses that require the nuclear host cell machinery for replication. Small DNA tumor viruses encode proteins able to induce resting cells to synthesize DNA thus overcoming the G1/S restriction point [8,9]. Herpesviruses in contrast arrest cells in late G1 phase or at the G1/S interface prior to host DNA synthesis [10,11]. Other viruses provoke a cell cycle arrest commonly associated to DNA-damage responses (DDR; [12,13]), implying multiple consequences for the virus life cycles (reviewed in [14]), including the escape from innate immune sensing as e.g. in HIV infections [15]. In spite of the important body of knowledge provided by these valuable studies, little is known on how cell cycle regulations and their perturbations are connected with the assembly and maturation of viruses.

To address this fundamental issue in a mammalian system, we investigated the effects of cell cycle regulations on the nuclear assembly of the parvovirus Minute Virus of Mice (MVM) infecting mouse and human fibroblasts. The members of the *Parvoviridae* have a ssDNA genome, are widely spread in nature [16,17] and their productive infection largely relies on host cellular factors [18,19]. Unlike small DNA tumor viruses, parvoviruses are unable to promote entry into the S phase, although their multiplication require basic factors of proliferative cells to convert the incoming ssDNA viral genome into a double-stranded DNA, which serves as template for transcription and genome replication [20]. The reaction of ssDNA convertion requires presumably DNA polymerase δ, the proliferating cell nuclear antigen (PCNA;[21]), plus other S phase-induced factors [22]. Another cell cycle dependent process of parvovirus infection is the upregulated transcription from the early promoter at the G1/S transition [23], which may contribute to onset the viral gene expression at early S phase [24]. As the infection progresses, most parvoviruses subvert the cell cycle eliciting a DDR as strategy to support viral replication [25–28], which arrest cells at the S or G2/M phases. Viral genome amplification, as well as hijacking cellular signaling and replication factors, require activities of the multifunctional non-structural (NS, Rep) parvoviral proteins [29,30]. However, it is unknown whether the cell cycle regulatory machinery, or the S-phase environment induced by the infection, controls parvovirus assembly and maturation.

Cell cycle dependence of viral life cycles is decisive for maturation and release of progeny virions. In parvoviruses, these factors have yet another importance as these viruses are currently being used in cancer and gene therapy trials [31,32], and as their pathogenesis is restricted to proliferative tissues [33]. Consequently the knowledge of the molecular mechanisms underlying productive parvovirus infection is required for determining target cells and efficient production of vectors for therapeutical applications. To this aim, mouse and human fibroblasts subjected to several growth arrests, were analyzed along cell cycle seeking control signals exerted on the nuclear translocation of parvovirus MVM structural subunits, capsid assembly, and virus maturation.

## Results

### Quiescent, G1, and G1/S arrested mammalian fibroblasts retain parvovirus capsid proteins in the cytoplasm

**Fig 1:**
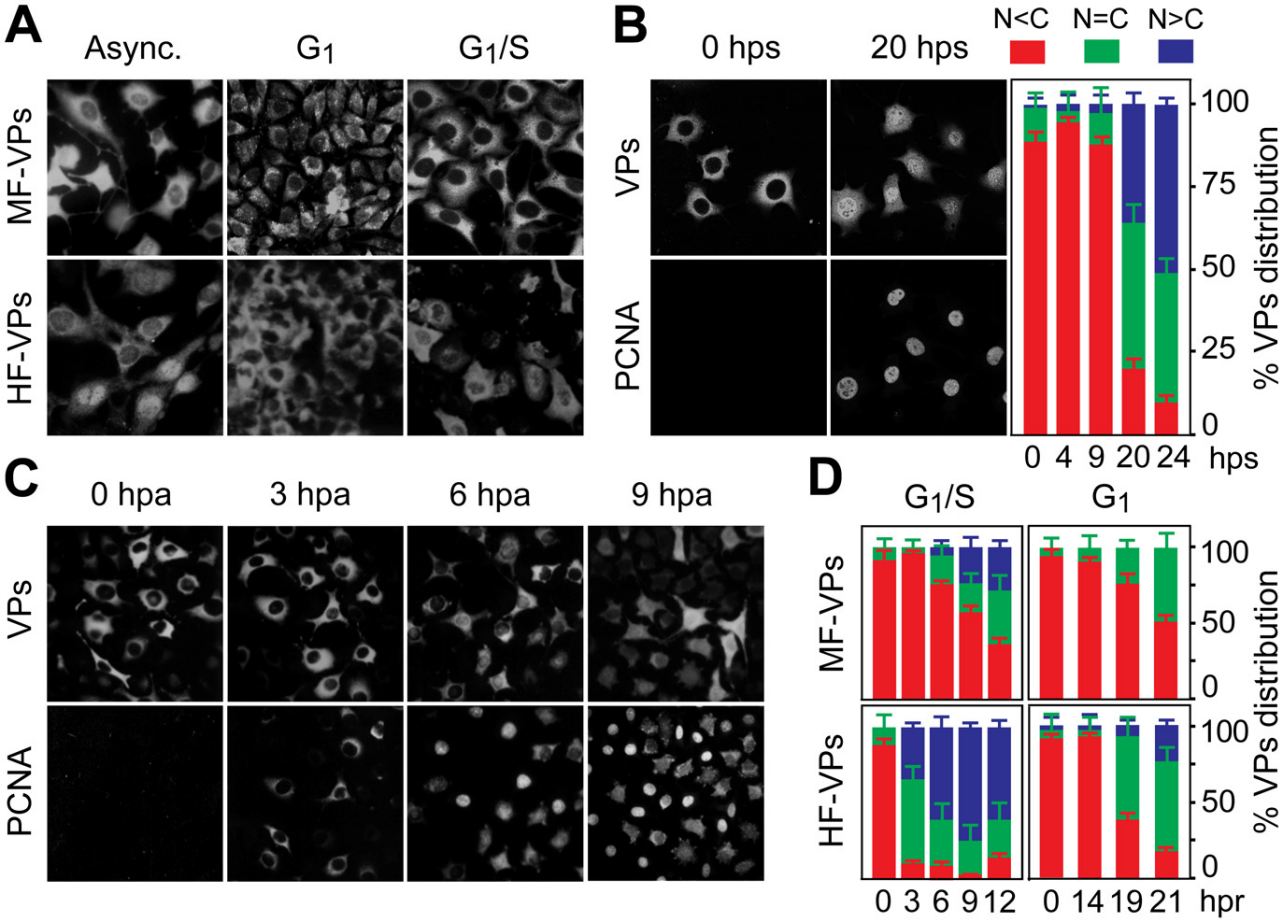
Cell cycle regulation of the nuclear translocation of MVM capsid proteins. **A**. MVM capsid proteins (VPs) are excluded from the nucleus at G0/G1. Microscopy analysis of mouse (MF-VPs) and human (HF-VPs) fibroblasts stably expressing VPs fixed as asynchronous cultures (async.), synchronized by density arrest (G1), or by isoleucine deprivation/aphidicolin (G1/S). **B**. Kinetic of VPs nuclear transport in quiescent (G0) mouse fibroblast induced into cycle by serum. *Left*, cells stained with the α-VPs and PCNA antibodies. *Right*, average percentages with standard errors from three experiments of VPs subcellular distribution at the indicated hps. **C**. VPs nuclear transport is allowed at S phase. PCNA and VPs subcellular distribution in synchronous MF-VPs at the indicated hpa. **D**. VPs phenotypes scored in mouse and human fibroblasts hours post-release (hpr) of synchronization by aphidicolin (G1/S), or by density arrest (G1). Bars represent average values with standard errors from three or four experiments. (N<C (red): predominantly cytosolic, N = C (green): equal distribution between cytoplasm and nucleus, N>C (blue): predominantly nuclear).

To investigate the regulation of MVM assembly by the cell cycle we first analyzed nuclear import of VP1 and VP2 capsid proteins (summarized as VPs) in the absence of other viral components. Pooled clones of transfected mouse or human fibroblasts stably expressing VPs (respectively named MF-VPs and HF-VPs) showed capsid proteins either predominantly cytoplasmic, or nuclear, or exhibited a homogenous stain by indirect immunofluorescence (IF) using the α-VPs antibody (Fig 1A, Async. panels), which mainly reacted with disassembled capsid subunits (see Materials and Methods), suggesting that VPs localization is affected by cell physiology. For further analysis, the transfected mammalian fibroblast lines were arrested by contact inhibition at high cell density (G1), or by isoleucine/aphidicolin (a DNA pol α inhibitor) double inhibition (G1/S), showing the VPs accumulated in the cytoplasm under both arrest conditions (Fig 1A). Such nuclear VPs exclusion was also observed in serum-starved quiescent MF-VPs (G0) (Fig 1B, 0 hps), collectively indicating that the nuclear translocation of the MVM capsid proteins is sensitive to different forms of cell growth arrests.

### Nuclear transport of parvovirus capsid proteins proceeds at the S phase of the cell cycle

To determine the phase of the cell cycle in which VPs nuclear translocation occured, their subcellular distribution was studied in a kinetic after cells were released from the three G0/G1/S arrest methods. PCNA, a well-defined regulator of DNA replication and cell cycle control [34],[21]), was used as cell phase marker. Firstly, the G0 to G1 transition-as defined in mouse fibroblasts [35]- was applied to quiescent MF-VPs. The VPs remained in the cytoplasm all along the G1 phase, up to 9 hours post-serum addition (hps), with less than 5% of the cells exhibiting a predominantly nuclear stain (Fig 1B, quantification). As cells entered S phase a mixed cytoplasmic/nuclear VP phenotype became apparent. By 20 hps, when increased nuclear PCNA staining indicated S phase, nuclear VPs accumulation was observable in 35% of cells, progressing to >50% at 24 hps (Fig 1B, 20 hps and quantification). To study VPs nuclear transport across G1/S transition under more stringent synchronization, mouse and human fibroblasts were synchronized by the isoleucine/aphidicolin double block. All cells showed intense cytoplasmic VPs stain with nuclear exclusion even at 3 hours post-aphidicolin release (hpa), correlating to the cytoplasmic PCNA observed in most cells (Fig 1C). At the S phase onset, which was indicated by a significant nuclear PCNA accumulation (6 hpa), 20% of the cells exhibited a homogeneous VPs stain, followed by increased nuclear VPs translocation when the cell cycle progressed to S phase (Fig 1C and 1D upper left). Noteworthy, the VPs translocation into the nucleus began earlier upon aphidicolin release and was more pronounced in HF-VPs than in MF-VPs (Fig 1D, *left*). For final confirmation, we analyzed VPs localization in cells released from the G1 phase caused by density arrest. Upon 14 hours post-subculture at low density (hpc) favoring S phase entry, cells showed a slow though progressive increase in VPs nuclear accumulation (Fig 1D *right*). However, a predominant nuclear phenotype was not observed in MFs, and, as after the aphidicolin treatment, the VPs nuclear transport was more efficient in HF-VPs than in MF-VPs. Collectively these synchronization studies demonstrated that the nuclear transport of VPs proceeds at the S phase of the mammalian cell cycle, at rates lower in mouse than in human transformed fibroblasts.

### S phase and the infection context govern parvovirus nuclear capsid assembly

**Fig 2:**
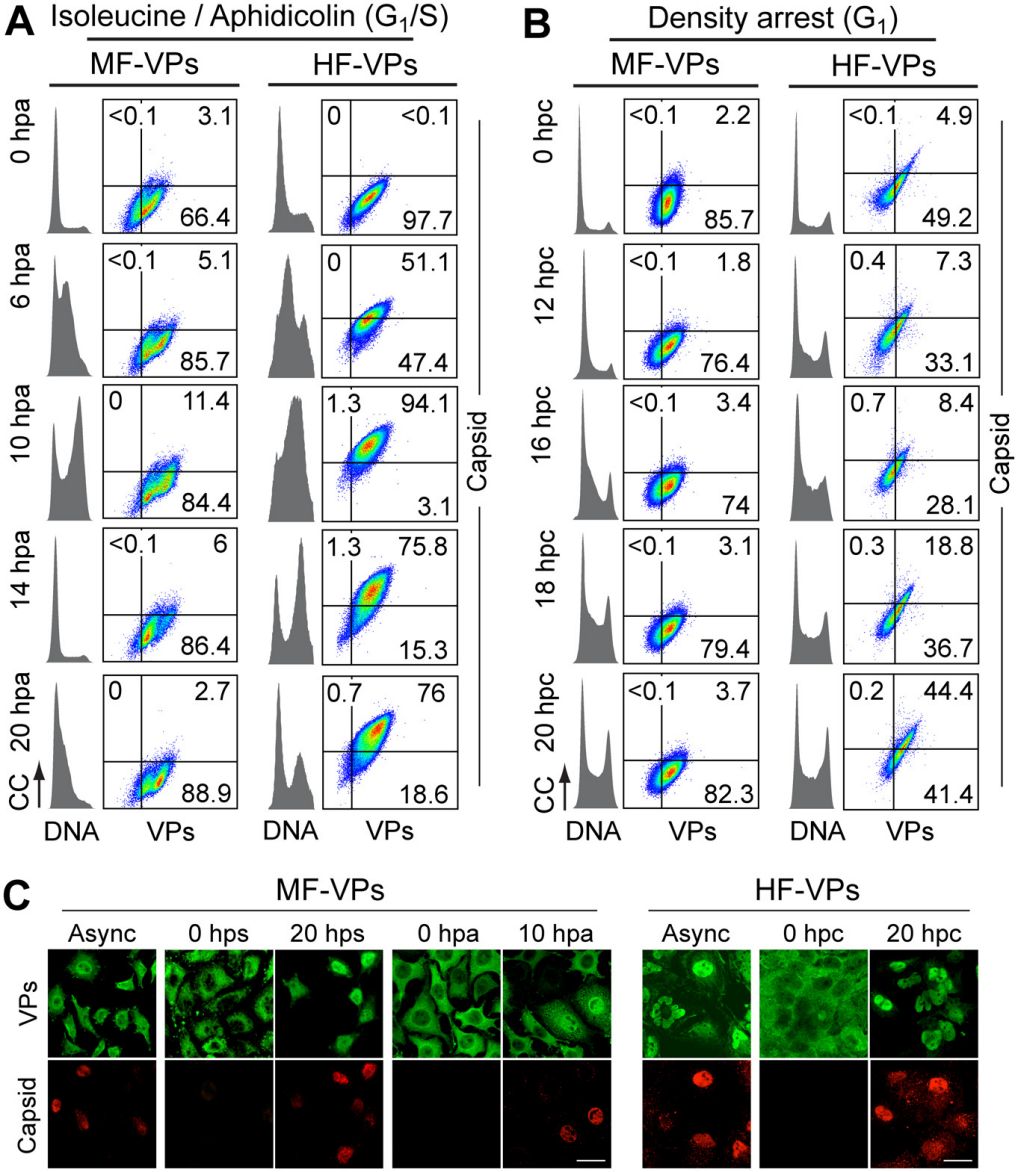
Cell cycle dependence of MVM assembly. Capsid formation and cell cycle progression in VPs stably expressing mouse (MF) and human (HF) fibroblasts. **A, B**: analysis by flow cytometry. Cells were synchronized by (**A**) isoleucine deprivation+aphidicolin (G1/S), or by (**B**) culturing to high density (G1), and stained for DNA content with DAPI (grey histograms), VPs and Capsid (biparametric dot plots), at the indicated hours post-arrest release (hpa or hpc, respectively). CC, cell count. The percentage of cells in the corresponding gates is shown. (**C**) Confocal analysis of the subcellular distribution of VPs and Capsid in VPs-expressing synchronous mammalian fibroblasts at the indicated hours post-release of the arrests. Scale bar 25 μm. Representative results from more than four independent experiments of each type are illustrated.

**Fig 3:**
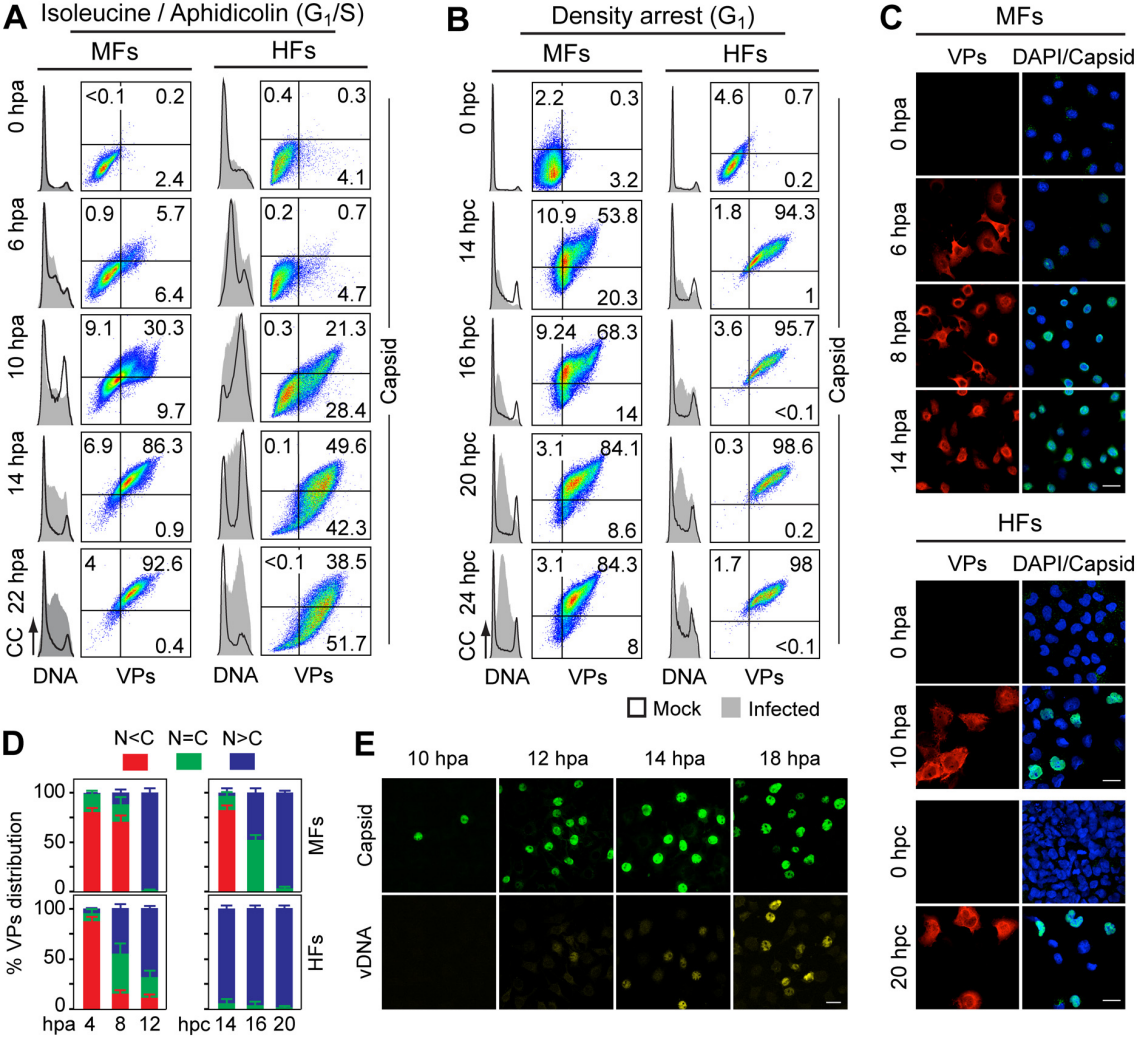
Cell cycle dependence of VPs expression and capsid assembly in the synchronous MVM infection of mammalian fibroblasts. VPs expression and capsid formation in infected mouse (MFs) and human (HFs) fibroblasts analyzed by flow cytometry **(A, B)**, or IF **(C, D)**. Cells were synchronized by (**A**) isoleucine deprivation+aphidicolin (G1/S), or by (**B**) culturing to high density (G1), and stained for DNA content with DAPI (histograms: blank for mock, grey-filled for infected), VPs and Capsid (biparametric dot plots), at the indicated hours post-arrest releases (hpa or hpc, respectively). The percentage of cells in the corresponding gates is shown. CC, cell count. (**C**) Nuclear transport and assembly of MVM capsid subunits in synchronously infected mammalian fibroblasts. The figure shows representative fields of VPs and Capsid localized by double IF in MVM infected mouse and human fibroblats at the indicated hours post-release of the isoleucine/aphidicolin (G1/S) or growth to confluence (G1) cell cycle arrests. Scale bar 25 μm. **(D)** VPs subcellular distribution in synchronously infected mouse and human fibroblasts at the indicated hours post-release of the G1/S (hpa) or G1 (hpc) arrests. Average values with errors from four experiments are shown. (**E**) Synchronously infected MFs at G1/S stained for assembled capsids and viral DNA synthesis by FISH-hybridization (vDNA) along hpa. The figure shows representative confocal fields of cells from three experiments. Scale bar 10 μm.

**Fig 4:**
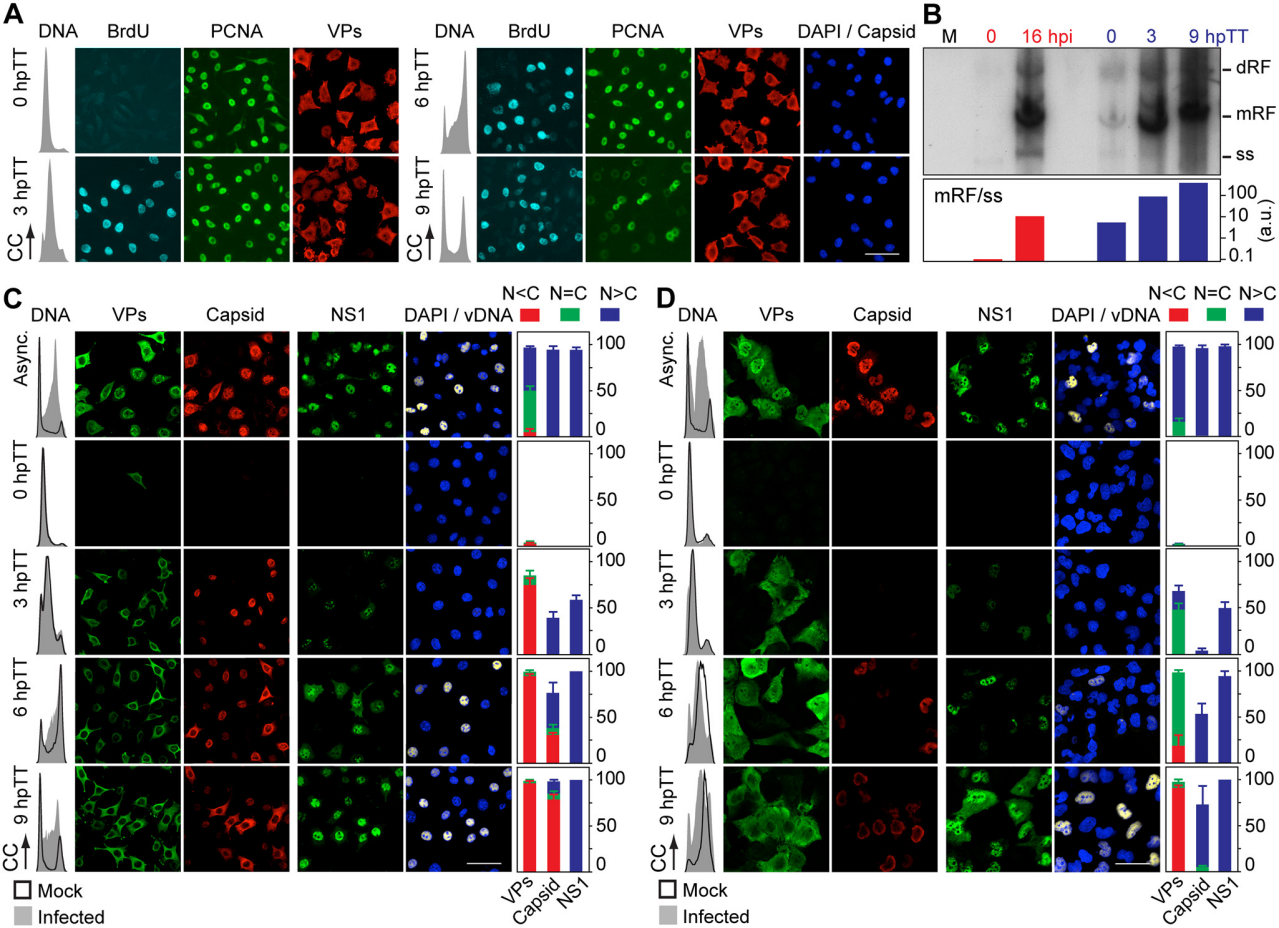
Stressing cellular DNA synthesis uncouples parvovirus genome replication from nuclear capsid assembly. **A**. Cellular DNA synthesis dependence of VPs subcellular distribution and assembly in transfected MFs. The figure shows the subcellular distribution of BrdU incorporation, PCNA, VPs, and Capsid in MF-VPs analyzed by confocal microscopy at the indicated hours post-release of the double thymidine block (hpTT). *Left*: histograms of cell cycle analysis by flow cytometric determination of DNA content. **B**. Stressing cellular DNA synthesis hampers the packaging of parvovirus genome. Southern-blot analysis (performed with a full-lenght ^32^P-MVM probe as described [42]) of viral replication and maturation in growing (hpi) and thymidine synchronized (hpTT) infected MFs. Low molecular weight DNA isolated from 10^5^, or from 5x10^5^ (in 0 hpTT and 3 hpTT) cells, was loaded per lane. M, mock infection. mRF and dRF, monomeric and dimeric viral replicative intermediates, respectively; ss, single-stranded viral genomes. Below, mRF/ss ratio of synthesis measured by densitometry. a.u, arbitrary units. DNA synthesis stress hampers parvovirus capsid assembly in infected MFs (**C)** and HFs (**D**). The subcellular distribution of VPs, assembled capsid (Capsid), NS1, and viral replication analyzed by FISH-hybridization (vDNA), is shown by confocal microscopy in synchronously infected mouse and human fibroblasts at the indicated hpTT. Async: non-synchronous cultures. DNA (left columns): cell cycle phases at the respective time post-release analyzed by cytometry (histograms: blank for mock, grey-filled for infected). Right bars: percentages with standard errors of subcellular distribution of the viral antigens. Scale bar in IF is 50 μm.

To investigate if the cell cycle may also regulates viral capsid formation, MF-VPs and HF-VPs cultures, synchronized by the double isoleucine/aphidicolin block (G1/S) or growth to density arrest (G1). We used flow cytometry (Fig 2A and 2B) and confocal IF (Fig 2C) applying the α-VPs and α-Capsid antibodies. The specificity of this α-Capsid monoclonal antibody to recognize an epitope only conformed in the assembled MVM capsid was previously characterized by biochemical [36–38], genetic [39], and structural analyses [40]. The constitutive expression of viral structural proteins did not affect the timing of cell cycle progression from G1 to G2/M in MF-VPs and HF-VPs (cell cycle histograms in Fig 2A and 2B) as compared to untransfected controls (Fig 3A and 3B; see also Fig 4A and 4C). The VPs retained in the cytoplasm of the fibroblasts arrested at G0, G1/S or G1 phases failed to assemble into capsids (Fig 2). While VPs expression remained essentially stable throughout the cell cycle, capsid formation in cells released from the G1/S arrest started when their DNA content increased denoting entry into S phase (Fig 2A). Progression through S phase was more reliable in cells released from G1/S than from G1 arrests, and occurred faster in MFs than in HFs (Fig 2A and 2B). Capsids were exclusively formed in the nucleus of transfected fibroblasts (Fig 2C) with efficiencies strictly correlated to the rate of VPs nuclear transport shown above (Fig 1). Thereby the proportion of MF-VPs showing assembled capsid was low in G1/S (Fig 2A) and not significant in cells released from the G1 arrest (Fig 2B), as compared with the efficient capsid formation in HF-VPs (Fig 2). Cells forming capsids reached a height at late S phase (Fig 2A, 10 hpa panels), and declined as the cells underwent mitosis (14 hpa panels). We therefore concluded that MVM capsid formation is a nuclear S phase dependent process.

For verification of these findings in the context of a productive viral infection, both mammalian fibroblast cell lines synchronously infected by MVM at G1/S and G1 were studied by flow cytometry (Fig 3A and 3B), and IF as read-out (Fig 3C). Synthesis of the VPs was only detected upon release of the arrests (aphidicolin or cell density), even though the virus was inoculated several hours earlier (see Materials and Methods), showing the tight control of parvovirus gene expression in growth-arrested cells. VPs gene expression and capsid formation was first detected in MFs and HFs concomitantly with their entry in S phase as indicated by an incfreased DNA content (Fig 3A; MFs: 6 hpa, HFs: 10 hpa). In both infected fibroblast cell lines released from the two arrest methods, the number of VPs expressing cells forming capsids raised along S phase, reaching a height as the cell cycles became arrested by the infection prior G2 (see S1A and S1B Fig for quantitative cell cycle data). Importantly, an efficient VPs nuclear translocation occurred in most infected cells across S phase (Fig 3D), which was followed by an immediate assembly in nuclear capsids (Fig 3C). This high level of nuclear capsid formation in synchronously infected MFs (Fig 3) was in sharp contrast to the poor capacity observed under similar conditions in MF-VPs transfected cell (Fig 2). Of note, the VPs transport and nuclear capsid assembly were accomplished within early-mid S phase, several hours prior the bulk of viral DNA replication (Fig 3E), which occurred once infected cells have developed a pseudo-S phase or S/G2 arrest (Fig 3A and 3B, S1 Fig).

### Cell cycle controls of parvovirus genome replication and capsid assembly are uncoupled

The need of S phase-related factor(s) affecting VPs nuclear transport led us to investigate if this process is supported by cellular DNA synthesis. MF-VPs were synchronized at G1/S by a double thymidine (a metabolic inhibitor) block (TT), and the VPs subcellular distribution was then analyzed during the DNA replication arrest and upon release. In arrested MF-VPs (Fig 4A, 0 hpTT panel), under cellular *de novo* DNA synthesis inhibition, which was shown by the flow cytometric analysis of DNA content and absence of BrdU staining, PCNA was accumulated in the nucleus as previously reported [41]. In contrast, the VPs showed mainly a cytoplasmic or mixed phenotype. When cells were released from the TT block and proceeded through S phase, which again was indicated by DNA content and synthesis activation (Fig 4A, 3 hpTT panel), nuclear PCNA stayed on at high levels, while the VPs remained mostly cytosolically. We thus conclude that although S phase is required, the activation of cellular DNA synthesis *per se* is not sufficient for causing VPs nuclear translocation. The cytoplasmic VPs phenotype became predominant as cells progressed through S (6 hpTT) and G2/M (9 hpTT), when PCNA staining declined and mitotic figures appeared (Fig 4A, right panels). In consistency with our findings shown in Fig 2, capsids also failed to assemble in transfected MFs under the same method of synchronization (Fig 4A, capsid panels). This experiment thus demonstrated that VPs nuclear transport operates by an S-phase dependent mechanism distinct to that of PCNA, and sensitive to the cellular DNA synthesis stress caused by thymidine.

We next investigated VPs translocation in the context of a TT-synchronous infection of mouse and human fibroblasts. Viral replication and gene expression were not observed during the DNA synthesis arrest (Fig 4C and 4D, 0 hpTT panels). Upon release of the TT block the number of VPs and NS1 expressing cells raised abruptly, in particular as cells entered and proceeded through S phase (Fig 4C and 4D, 3 hpTT panels). Noteworthy, as observed above (Fig 3E), the peak of viral DNA synthesis in both cell types occurred hours delayed with respect to VPs expression, reaching a height at 9 hpTT (Fig 4C and 4D, vDNA panels to the right). At this time the cycle of most cells showed a pseudo-S phase or drastic arrest at S/G2 caused by the MVM infection (Fig 4C and 4D, DNA content plots to the left; quantitative analyses in S1C Fig). At early time post-release most infected MFs exhibited a cytoplasmic/mixed VPs phenotype that correlated to a significant nuclear capsid staining (Fig 4C, 3 hpTT). At late S phase however the VPs proteins became increasingly retained in the cytoplasm, paralleling the phenotype observed in MF-VPs, and assembled in cytoplasmic capsids in a high proportion of cells (Fig 4C, 6 hpTT). Consistently this phenomenon gained robustness at 9 hpTT, as we observed a predominant cytoplasmic VPs phenotype and a highly efficient mislocalized capsid in most cells, under a severe S/G2 arrest (Fig 4C, lower panels, and S2 Fig). The cytoplasmic capsid assembly occurred at the time when cells were undergoing nuclear viral genome replication (Fig 4C, vDNA panels), which suggests that the virus is unable to encapsidate the genome and mature in TT synchronized cells. Accordingly, progeny single-stranded viral genomes were not resolved in MFs released from TT arrest under the accumulation of high levels of viral DNA replicative intermediates (Fig 4B)

Like in MFs, thymidine arrest of HFs irreversibly impaired VPs nuclear accumulation. At 3 hpTT we observed as a predominant mixed phenotype which was followed by increasing cytoplasmic retention when the infection proceeded to later hpTT (Fig 4D, left panels). Capsid formation in HFs started since 6 hpTT but become apparent in most cells at 9 hpTT. However, the level of assembled capsids was very low if compared to that of asynchronous infections (Fig 4D, Capsid panels). Unlike the cytoplasmic assembly found in MFs, capsid formation was confined within the nucleus of the HFs at 9hpTT (S2 Fig). We assume that the delayed capsid formation in HFs with respect to MFs may be caused by the slower progression across S phase in HFs (Fig 4C and 4D, 3–9 hpTT; and S1C Fig), whereas the distinct subcellular distribution of the capsid assembly process suggests the implication of cell-type specific factors. Noteworthy, the lasting impairment of VPs transport caused by TT in both mammalian fibroblasts did neither perturb NS1 nuclear accumulation nor viral DNA synthesis to normal levels (Fig 4C and 4D, right columns of panels). In summary these data demonstrate that nuclear capsid assembly and viral genome replication respectively require early and late S phase cellular factors. We further conclude that both steps are mediated by independent mechanisms, as only the nuclear translocation of capsid subunits was irreversibly hampered by a DNA synthesis stress.

### Cell contact signals exerted on proliferating cells deregulate subcellular parvovirus assembly

**Fig 5:**
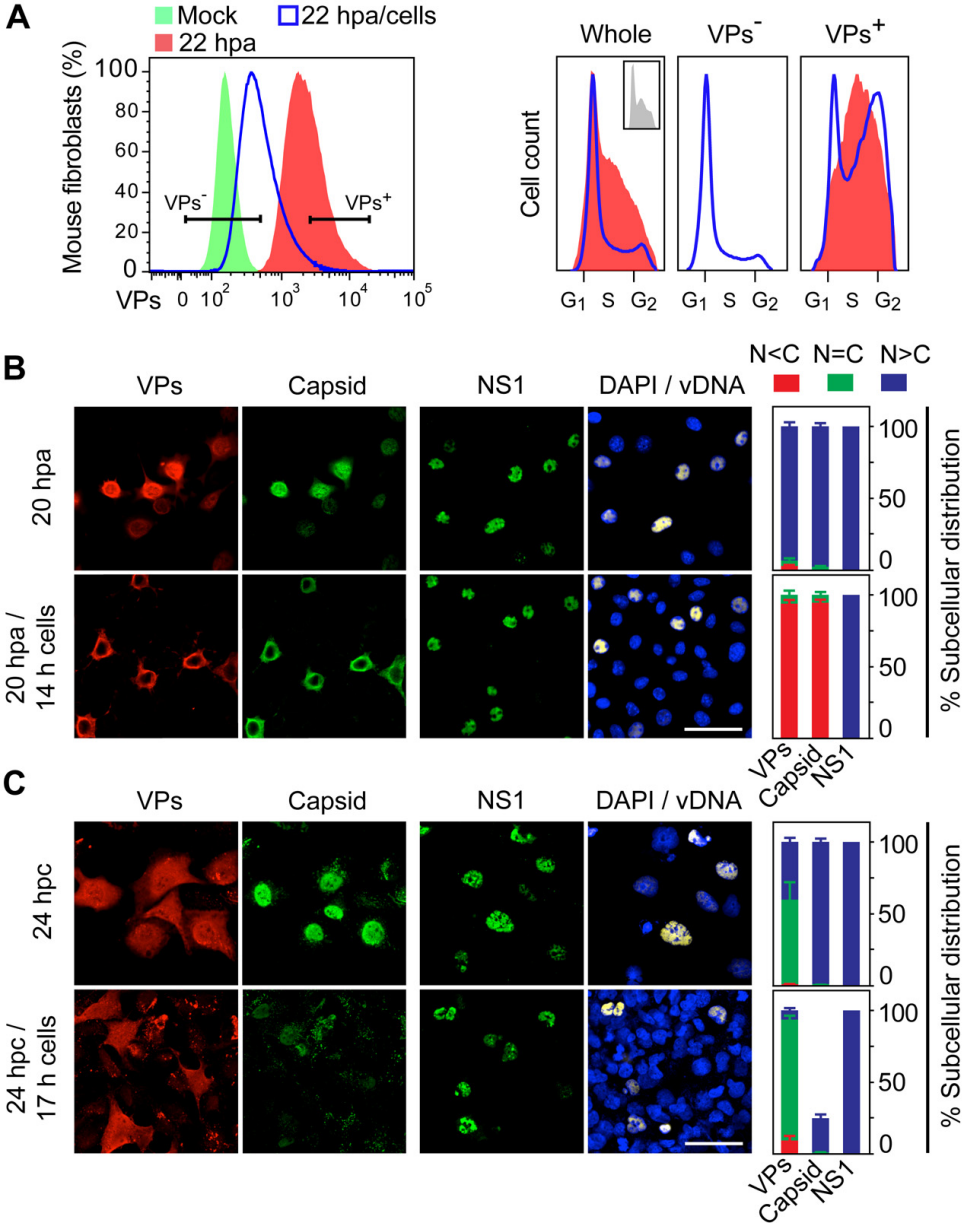
Cell density arrest signals perturb and may misplace MVM assembly. **A**. Flow cytometry of G1/S synchronously infected MFs sampled at 6 hpa, 22 hpa, or at 22 hpa but with density-arrest imposed since 6 hpa by uninfected exogenous cells (22 hpa/cells), analyzed for VP expression (left), and for cell cycle distribution (cell count vs. DNA content) of non-expressing (VP^-^) and capsid proteins expressing (VP^+^) cells (right). VP^-^ cells were not detected in 22 hpa samples. Inset: cell cycle distribution at 6 hpa. **B**. Confocal analysis of MFs synchronously infected by MVM showing the subcellular localization of viral antigens (VPs, Capsid, NS1), and viral genome replication (vDNA), at 20 hpa without or upon the previous addition of a saturating number of cells at 6 hpa (20 hpa/14 h cells). **C**. Similar analysis performed in HFs synchronously infected by density arrest without (24 hpc), or upon exogenous cell addition at 7 hpc (24 hpc/17 h cells). Scale bars 50 μm. The percentage of subcellular distribution of the viral antigens within the respective infected cell populations (VP^+^ staining) is shown on the right.

We next asked whether VPs nuclear transport and capsid formation in infection is sensitive to other cell growth controls, as cellular contact inhibition, which is fundamental in the homeostasis of multicellular organisms. To mimic this physiological process in cell culture, infected MFs highly synchronized at G1/S by aphidicolin were first released from the arrest to allow cellular and viral cycles progressions (see above), and when cells had entered S phase at 6 hpa (Fig 3A, left column), an excess of growing uninfected homologous cells was added to impose general cell contacts (see scheme in S3A Fig). As shown in Fig 5A, at the end of the normal infection cycle in control cultures (22 hpa), the cells expressing VPs to higher levels were at pseudo-S phase or arrested at S/G2 (Fig 5A, right). In density arrest cultures (Fig 5A, 22 hpa/cells), while VP^-^ cells were mostly asynchronous due to the contribution of the growing uninfected exogenous population, the high VP^+^ cells had also progressed to the S/G2 arrest, even though a significant proportion (close to 30%) remained at G1. Thus, contact inhibitions developed in infected cultures at S phase allowed high levels of viral gene expression, even in cells failing to progress through the cell cycle.

Confocal microscopy analysis under this experimental setting (Fig 5B) showed a remarkable cytoplasmic retention of the VPs in the infected MFs brought to confluence (20 hpa/14h cells). This was in contrast to their predominant nuclear accumulation in control infections maintained at low density (20 hpa). The impaired VPs nuclear transport in infected cultures brought to confluence led to an exclusive and efficient cytoplasmic capsid formation (Fig 5B), which also occurred at other time of density arrest (S3A Fig lower panels). A parallel analysis performed in HFs brought to confluence at 7 hpc (scheme in S3B Fig upper), also showed a significant cytoplasmic retention of VPs in confluent cultures, which resulted in poor nuclear capsid formation in a low proportion of the infected cells (Fig 5C, quantitative data shown to the right). Noteworthy, in both MFs and HFs, nuclear accumulation of NS1, which is the major replicative viral protein, was not impaired by the density contact signals, neither was the synthesis of viral genomes (Fig 5B and 5C, right columns; S3A and S3B Fig lower panels). We conclude that cell contact signals irreversibly deregulate MVM maturation by hampering (HFs) or misplacing (MFs) capsid assembly.

### Cell cycle control of MVM assembly proceeds through oligomerization of phosphorylated VPs and is mediated by unconventional transport routes

**Fig 6:**
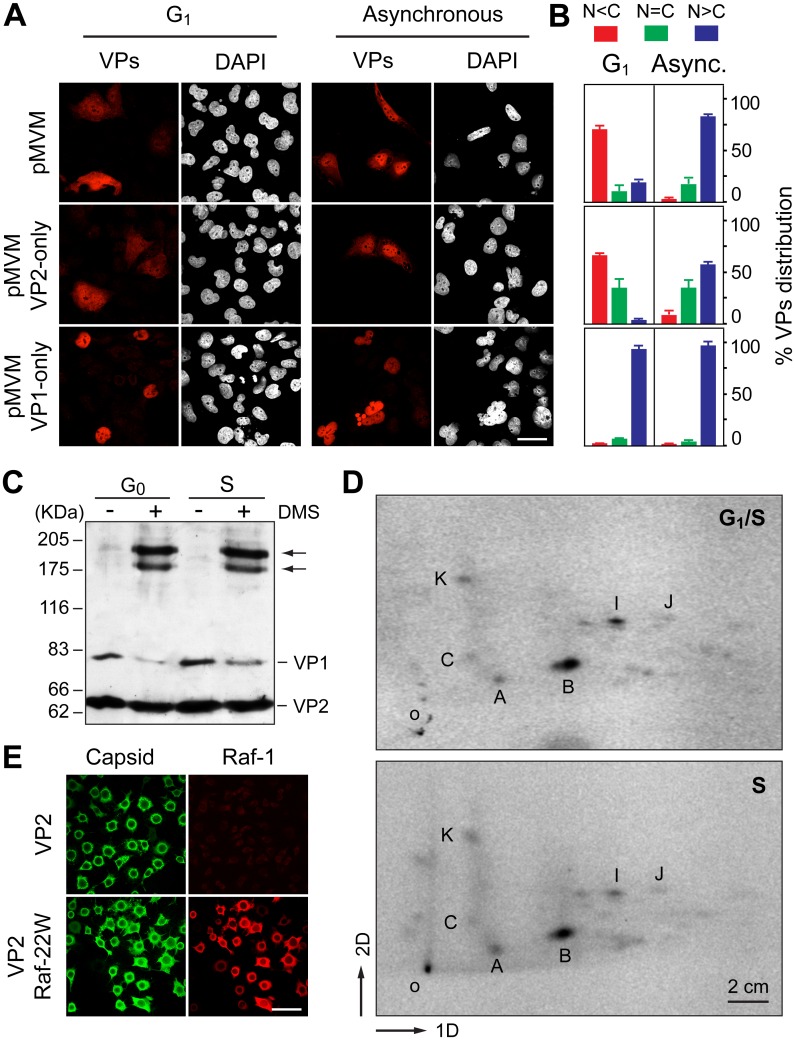
Signals and phosphorylations regulating the nuclear transport of MVM trimeric capsid assembly intermediates. **A**. Cell cycle dependence of VP1 and VP2 nuclear accumulation. Confocal images of HFs seeded at high (G1 arrest) or low (asynchronous growth) density, stained with the α-VPs serum upon 48 h postransfection with the indicated genomic MVM plasmids. Scale bar, 25 μm. **B**. Phenotypes of the VPs subcellular distribution for the indicated plasmids 48 h postransfection in HFs seeded at high (G1 arrest) or low (asynchronous growth) density, stained with the α-VPs antibody based on four independent experiments. **C**. The VPs assemble into trimers in the cytoplasm of growth arrested mouse cells. Homogenates of MF-VPs made quiescent by serum starvation (G0), or upon 8 h post-stimulation into cycle by serum addition (S), were cross-linked with DMS and analyzed by 5% SDS-PAGE and Western-blot (α-VPs antibody). Arrows indicate the two types of VP trimers [37]. **D**. Pattern of VP2 phosphorylation in the G1 to S transition. 2D-tryptic phosphopetides analysis of the VP2 protein purified from HF-VPs, ^32^P-labeled either during isoleucine/aphidicolin arrest (G1/S), or 10 h upon arrest release (S). Phosphopeptides were monitored by phosphoimaging and are named as previously reported [43]. 1D, 2D: first and second dimensions; o, origin. **E**. Raf-1 phosphorylated VP2 fails to translocate into the nucleus of insect cells. H5 monolayers infected by the Bac-VP2wt alone (upper panels), or co-infecting with the Bac-Raf1-22W (lower panels) baculovirus, stained at 24 hpi with the α-capsid and α-Raf1 antibodies. Scale bar 50 μm.

To get insights into the molecular mechanisms involved in the cell-cycle dependent VPs nuclear translocation, we analyzed the nuclear transport of each capsid protein (VP1 and VP2) individually expressed from MVM genomic clones. VP2 nuclear translocation is driven by a so-called structured nuclear localization motif (NLM) [36], whereas the NLM of singly expressed VP1 subunits can be assisted for transport by conventional NLSs at its specific N-terminal sequence [36,42]. Transfected HFs seeded at high density to provoke growth arrest in G1 showed significant cytoplasmic retention of VP2 (Fig 6A and 6B, middle panels). In sharp contrast, VP1 was translocated into the nucleus efficiently and independently of cell growth conditions (Fig 6A and 6B, lower panels). When both proteins were co-expressed in growth arrested cells, the VPs were also significantly retained in the cytoplasm, which was not the case in growing cells (Fig 6A and 6B, upper panels). This observed difference was independent upon the MVM strain used to derive the plasmid mutants (see Materials and Methods). Since a functional NLM was required for the efficient transport of both VP subunits [42], the result shown in Fig 6A and 6B suggests that the NLM motif dominates over the NLSs of VP1, driving VP2 and VP1/VP2 complexes into the nucleus by a mechanism sensitive to density-arrest signals.

The role of VPs oligomerization and post-translational modifications during their S-phase dependent transport was next evaluated. In quiescent and S-phase induced MF-VPs, the VPs could be cross-linked with similar efficiency into oligomers (Fig 6C). They correspond in their size to the two types of trimers (2VP2/1VP1, and 3VP2) which were found as the major capsid assembly intermediates in the infection [37]. Both types of trimers also assembled in HF-VPs (S4 Fig) at times post-release of G1 during which VPs predominantly localized in the cytoplasm (Fig 1D). These results indicate that the cell cycle arrest did not inhibit the cytoplasmic formation of VPs trimers, which is required for their nuclear transport [37]. We next studied the phosphorylation pattern of the VP2 subunits, which is the major component of the capsid assembly intermediates, at distinct cell cycle phases. Phosphorylation at Ser residues by the Raf-1 kinase of the MAPK pathway was shown to be required for VP2 nuclear import [38]. VP2 proteins isolated from the cytoplasm of HF-VPs arrested by aphidicolin at G1/S, or from the nucleus at 10 h post-release (S phase), showed an identical 2D tryptic phosphopeptides map (Fig 6D). This pattern was similar to that found in the MVM infection [43] and VP2 phosphorylation by Raf-1 *in vitro* [38]. These data suggested that cytoplasmic phosphorylation of VP2 by Raf-1 is required, but may not be sufficient, to explain the S-phase dependent nuclear import of the VPs trimers.

**Fig 7:**
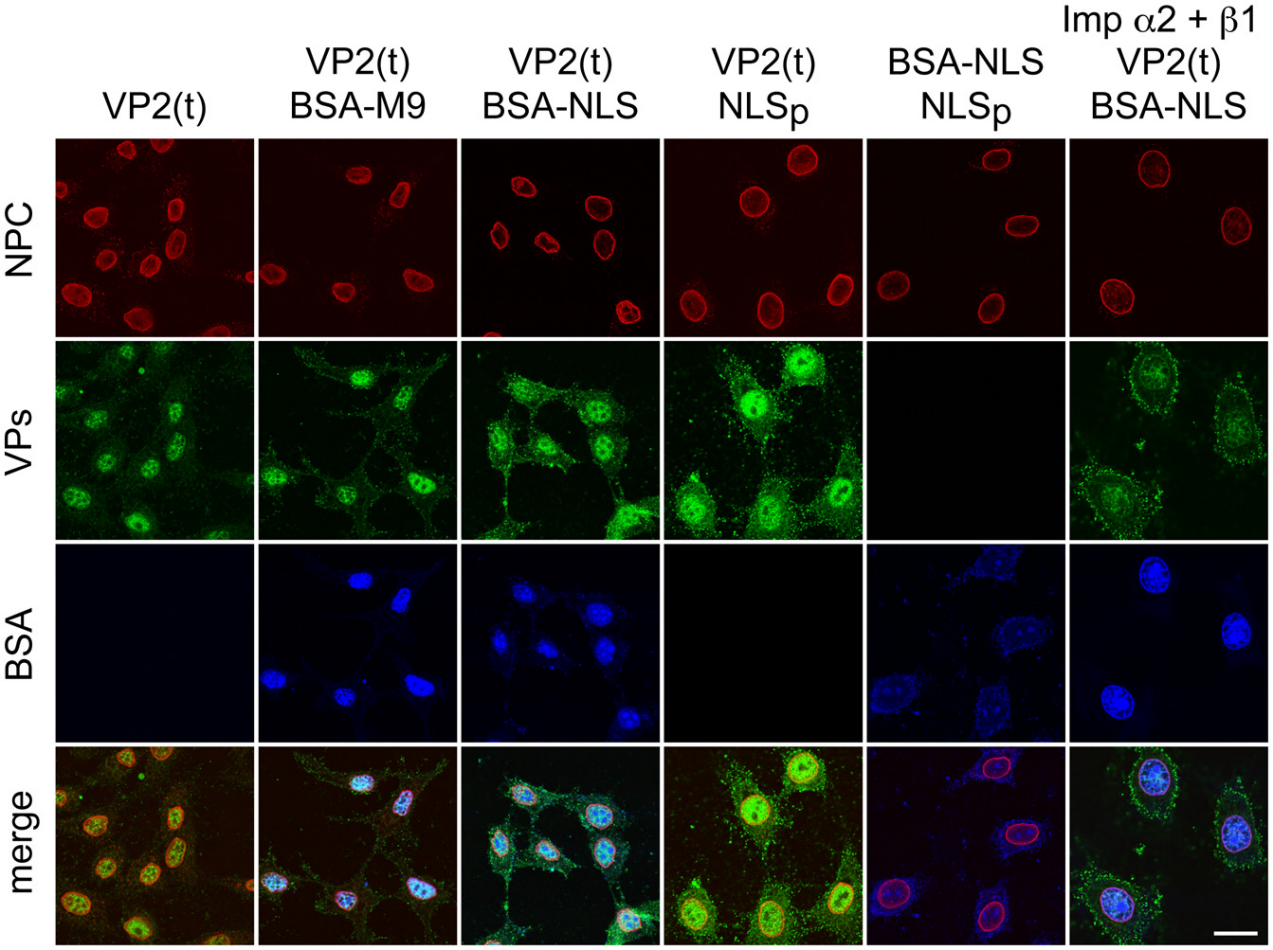
Importins α/2β1 and transportin are not involved in VP2 nuclear import. Transport of VP2-trimer (t) analyzed by confocal microscopy in Digitonine-permeabilized HeLa cells, in combination with competitor markers. The transport phenotype of 200 ng of gradient-purified VP2(t) in the presence of 10 μg of BSA conjugated to peptides of characterized import routes (BSA-NLS and BSA-M9), or of 3.5 μM of NLS peptide alone (NLSp), are shown. Transport factors were provided by rabbit reticulocyte lysates (first five columns) or by purified importins (right columns). Representative results from three experiments are shown. Scale bar 10 μm.

We finally addressed the import pathway used by the VP2 trimer (t). In a heterologous insect system, VP2 assembled into capsid but failed to enter the nucleus alone or upon co-expressed with a constitutively active form of Raf-1 (Fig 6E), even though the VP2(t) isolated from insect cells under these experimental conditions was phosphorylated by Raf-1, and this post-translational modification sufficed VP2(t) for nuclear transport in human cells [38]. This experiment thus suggested that, unlike multiple nucleocytoplasmic transport routes [44], [45], the cellular machinery transporting VP2(t) into the nucleus is not evolutionary conserved. To study the transport pathway relevant for the MVM infection of mammalian cells, phosphorylated transport competent VP2(t) purified from HFs transfected at large scale, was used as substrate for an *in vitro* transport assay in digitonin-permeabilized HeLa cells. We analyzed the need of nuclear import receptors of the karyopherin (importin) α/β system binding classical NLSs [46,47], and of the transportin (Kap β2) translocating M9 domain-exposing proteins (as representative of the PY-NLS sequences) [48,49]. VP2(t) were efficiently translocated into the nucleus when a rabbit reticulocyte lysate (RRL) was used as source of transport factors (Fig 7, left column). The import of VP2(t) into the nucleus though could not be blocked by tenfold molar excess of either BSA-NLS or BSA-M9 marker conjugates (Fig 7, second and third columns). Consistently, a fifty fold molar excess of a NLS peptide failed to compete VP2(t) import, while the import of BSA-NLS was inhibited (Fig 7, fourth and fifth columns). In agreement with these observations, a replacement of the cytosolic RRL extract by Ran-GTP and purified α2 and β1 importins failed to translocate the VP2(t), despite of an active nuclear transport of the BSA-NLS cargo using the same conditions (Fig 7, right column). These results thus indicate that the phosphorylated VP2(t) access a route(s) distinct from both the importins α2/β1 and the transportin pathways for its cell cycle dependent nuclear import.

## Discussion

### Cell cycle regulated nuclear transport and assembly of parvovirus capsid subunits

Our study shows an unprecedented cell cycle regulation of viral nuclear assembly. The capsid proteins of the parvovirus MVM were excluded from the nucleus of mammalian fibroblasts as quiescent (G0), arrested at G1, or at G1/S boundary, but they were translocated into the nucleus upon release of the arrests, at efficiency depended upon the inhibitor and cell type (Fig 1). VPs nuclear import was not directly stimulated by the numerous serum responsive genes immediately induced by growth factors at the G1/S restriction point [35,50], since it only proceeded as transfected and infected fibroblasts traversed through S phase (Figs 1–3). Putative cellular factors induced at S phase were sufficient to provoke high VPs nuclear transport in HFs (Figs 1 and 2), but failed to do so in MFs, the latter requiring MVM infection (Figs 1–3). A major factor contributing to the different efficiency of transport between both transfected fibroblast cells may be the level of VPs phosphorylation, a modification essential for efficient VP2 nuclear targeting catalyzed by the Raf-1 kinase, which is part of the MAPK signaling cascade ([38]; see also below). In agreement with this assumption, the endogenous Raf-1 kinase activity was found to be much lower in MFs than in HFs [38].

**Fig 8:**
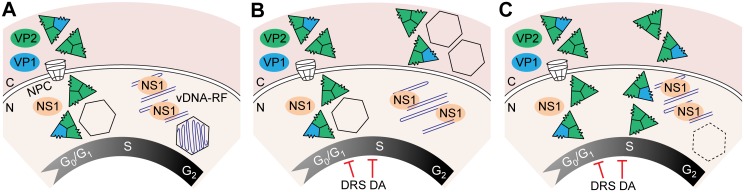
Temporal course of functional and disrupted cell cycle control on parvovirus assembly. **A**. Parvovirus assembly and maturation is timely coupled to cell cycle progression from G1 to G2. Phosphorylated VPs trimers translocate into the nucleus at early S phase driven by the NLM, and assemble into empty capsids. Viral genome replication (vDNA-RF) and virions maturation occur as cells become arrested at the late S/G2 phases by the infection. Infected cells subjected to thymidine DNA replication stress (DRS), or density arrest by cell contacts (DA), interrupt drastically VPs nuclear transport, resulting into cytoplasmic empty capsids formation in MFs (**B**), or inefficient nuclear assembly in HFs represented by dotted capsid (**C**). Acronyms: C, cytoplasm; N, nucleus; NPC, nuclear pore complex.

The VPs translocated into the nucleus of the mammalian fibroblasts immediately assembled into capsids at S phase. Accordingly, capsid formation efficiently occurred in HF-VPs traversing S phase, whereas MF-VPs showed a limited capacity (Fig 2). The efficient capsid forming capacity of infected MFs (Fig 3) suggests that expressed viral factor(s) contribute to VPs nuclear transport. This activity may well be mediated by the NS2 protein, which was dispensable for the MVM infection of human transformed fibroblasts but required for MVM capsid assembly in MFs ([51] and references therein). In the synchronous infection therefore, gene expression, VPs nuclear translocation, and capsid formation, occurred at early-mid S phase, several hours prior the accumulation of viral DNA replicative intermediates at the late pseudo-S phase arrest (Fig 3). As illustrated in Fig 8A, this timely orchestrated virus life cycle across S phase implies that gene expression results from the input ssDNA genomes converted to dsDNA templates or from basal levels of replicated viral genomes. A timely dissociated use of templates for transcription and for DNA amplification is commonly found in virus life cycles, and may have evolved also to maximize genome encapsidation into preformed capsids.

### VPs nuclear transport and cellular DNA synthesis

The cellular DNA synthesis was identified as one major S-phase process contributing to the control of VPs nuclear transport. The VPs retained in the cytoplasm by the G1/S aphidicolin arrest translocated into the nucleus of transfected fibroblasts at S phase concomitantly with PCNA translocation (Fig 1). However, inhibition of DNA synthesis by thymidine hampered VPs transport although allowing efficient PCNA nuclear accumulation (Fig 4A), as did other cellular DNA synthesis stresses [41]. The differential transport requirements of VPs and PCNA may reflect their specificity of interacting partner proteins. PCNA nuclear accumulation was regulated by export signals [52] and a non-classical NLS segment directly binding importin-β [53], whereas classical NLSs and importin-β were not used by the VPs (Figs 6 and 7). Moreover, restored cellular DNA synthesis upon thymidine release inhibited VPs nuclear transport across S-G2 phases of infected mammalian fibroblasts (Fig 4C and 4D). This was in sharp contrast with the unrestricted transport at early-mid S phase of infected cells released from the aphidicolin arrest (Fig 3). The basis of the distinct sensitivity of VPs transport to the DNA pol α inhibition by aphidicolin or the metabolic inhibition by thymidine remains unclear. Putative mechanisms could involve a decay of transient transport factors provoked by the altered S phase progression that follows thymidine inhibition [54]. The availability of transient factors for viral VPs transport would also be more restricted upon thymidine than aphidicolin arrests because the substantial shortening of the S phase length in TT-treated cells (compare Figs 4C and 4D vs 3A, and also S1C vs S1A Fig). Even though the involved mechanisms remain unknown, the subcellular distribution of viral antigens and the levels of viral DNA amplification shown in Fig 4B–4D demonstrate that VPs nuclear transport is most sensitive to cellular DNA synthesis stresses. This implies that this step is the bottleneck of parvovirus life cycle, which may largely influence the biology of these viruses in nature.

### Perturbing cell cycle progression inhibits or misplaces parvovirus capsid assembly

The cell cycle regulations also determined the subcellular compartment of parvovirus capsid assembly. While transfected and infected cells normally form MVM capsids in the nucleus (Figs 2 and 3), infected fibroblasts subjected to cell cycle perturbations—here exemplified by a DNA replication stress caused by thymidine (Fig 4), and by exogenous density-contact signals (Fig 5)—abruptly impaired capsid assembly. In HFs these cycle perturbations inhibited assembly, although allowed low levels of nuclear capsid formation both at 6–9 hpTT (Fig 4D and S2 Fig) and in density-arrested cells (Fig 5C and S3B Fig). In MFs some nuclear capsid formed only at 3 hpTT (Fig 4C). The different hpTT at which capsids preferently assembled in HFs and MFs may relate to their characteristic timings of G1–G2 cell cycle progression (Fig 4C and 4D, S1C Fig), which would affect the availability of early-mid S phase transient transport factors (suggested above). However both cell cycle perturbations eventually led in infected MFs to a remarkable misplaced cytoplasmic capsid assembly (Figs 4C and 5B; S2 and S3A Figs), reflecting the capacity of these mouse cells to form MVM capsids regardless the subcellular compartment of VPs accumulation. Moreover, none of the assayed conditions inhibiting or misplacing capsid assembly altered other key cell cycle dependent viral functions, like high levels of gene expression, genome replication, or NS1 nuclear accumulation (Figs 4B–4D and 5, S3 Fig). The Fig 8 illustrates the two types of virus assembly defects observed in fibroblasts subjected to cell cycle perturbations (DNA synthesis stress and density-arrest): either accumulation of empty capsids in the cytoplasm of MFs (Fig 8B), or poor nuclear capsid formation in HFs (Fig 8C). In natural infections, an inhibited or misplaced capsid assembly may critically hamper parvovirus maturation and spreading in cells with stressed DNA replication conditions or dense cell-to-cell contacts, thereby restricting their pathogenesis, therapeutic efficacy, or favoring persistence in host tissues.

### Signals and mechanisms controlling the cell cyle regulated VPs nuclear transport

The nuclear transport of VP1 and VP2 capsid subunits showed different sensitivity to cell cycle controls. VP1 transport was not hampered by cell-density arrest signals (Fig 6A and 6B, bottom panels), suggesting that the NLS and other entry functions on its N-terminal domain [42,55,56] still allow the delivery of the incoming viral genome into the nucleus of quiescent and growth arrested cells. This is consistent with classical reports on the capacity of parvoviruses to infect resting and proliferating cells with similar efficiencies [57–59]. However, VP2 and VP2/VP1 complexes transport responded to cellular growth arrest signals (Fig 6A and 6B), which is based on cytoplasmic trimeric assembly intermediates (Fig 6C and S4 Fig). This supports that the 3VP2 and 2VP2/1VP1 trimeric assembly intermediates of MVM [37] follow a transport pathway sensitive to signals regulated by the cell cycle, and which are distinct from that of VP1. A nonconventional cell cycle-dependent nuclear import route accessed by the two types of VPs trimers is in agreement with previous findings on a structured NLM motif driving the process [36], substantially distinct from the disordered conventional NLSs [46,47,49], which interact with evolutionary conserved transport receptors of the importin/karyopherin family [60]. Consistently, permeabilized cells showed nuclear import of VP2 trimers independent of importin (NLS) and transportin (M9) (Fig 7), and the transport route accessed by the Raf-1 phosphorylated VP2 trimer in growing human cells [38] was non-conserved in insects (Fig 6E), unlike the common evolutionary conservation of multiple conventional nucleocytoplasmic transport mechanisms and karyopherins functions across the Eukaryota [44,45]. The NLM configuration at the internal capsid surface [36] would explain that capsids remain in the cytoplasm of infected mouse fibroblasts when assembly is misregulated (Figs 4C, 5B and 8B, S2 Fig). Whether this restriction on VPs nuclear transport and capsid assembly plays a role in parvovirus (and other nuclear viruses) host range deserves further investigations.

A plausible molecular explanation of the cell cycle-dependent parvovirus assembly may rely on the finding that VP2 nuclear translocation required specific phosphorylation by the Raf-1 kinase [38] of the MAPK cell cycle regulatory pathway [61]. Signaling in density arrested cells also involves permanent or pulses of ERK activation [62], which is phosphorylated by MEK in the cytoplasm, a main target of Raf-1 [61]. Thus, VP2 phosphorylation and oligomerization into nuclear transport competent trimers would enable the MVM assembly process to be coupled to subtle signals promoting cell cycle progression and proliferation. However VPs trimers containing VP2 subunits specifically phosphorylated by Raf-1 accumulated in the cytoplasm of growth arrested human fibroblasts (Fig 6D), suggesting that other modifications affecting the configuration of the trimers, or the concourse of an S phase-dependent transport machinery, may be required for VPs nuclear import. Interestingly, other Raf-1 interacting partners harbouring regulatory properties on the cell cycle also fail to immediately translocate into the nucleus ensuing phosphorylation. For example, the physical interaction of Raf-1 to the retinoblastoma tumor suppressor protein (Rb) at early G1 regulates its function and initiates a phosphorylation cascade required for cell cycle progression through the G1/S boundary [63,64], suggesting that further research on factors mediating parvovirus assembly may unravel major regulators of the mammalian cell cycle.

## Materials and Methods

### Mammalian cells

The A9 ouab^r^11 mouse fibroblasts, a variant of the mouse L cells (kindly provided by Jean Rommelaere, DKFZ, Heidelberg), and the NB324K simian virus 40–transformed human newborn kidney fibroblasts (kindly provided by Bernard Hirt, Epalinges, Switzerland) cell lines described as hosts for the MVM strains [43,65,66], as their respective derived transfected pools of clones (HF-VPs and MF-VPs, see below), were maintained under minimal number of passages in Dulbecco modified Eagle medium (DMEM) supplemented with 5% heat-inactivated fetal calf serum (FCS; Gibco). The Hela human cell line (ATCC: CCL2) was used in transport assays.

### Cellular synchronizations

The following synchronization methods were used for mouse and/or human fibroblasts as indicated. In all cases synchronization and cycle progression were controlled by DNA content in flow cytometry. G0, quiescence: mouse fibroblasts (A9 cell line and MF-VPs) were seeded (1.5x10^5^ cells/P60 mm dish) in DMEM-5% FCS for 16 h, washed several times with PBS, and then incubated in DMEM-0.1% FCS for 60–72 h. Cells were induced into cycle replacing the medium for fresh DMEM supplemented with 20% FCS [35]. G1, density arrest: mouse and human fibroblasts were seeded to a density of 5x10^5^ cells/P60 dish in DMEM-5% FCS. The medium was replaced daily to avoid acidification and after 72 h the cultures had reached confluence and arrested in G1 [67]. Cells were then set into cycle by sub-culturing seeding at low density (usually 2x10^5^ cells/P60 mm dish) in DMEM-5%FCS. G1-S, aphidicolin: mouse fibroblasts were para-synchronized either culturing in low serum (as above), or in isoleucine-free DMEM [68] supplemented with 5% dialyzed FCS. After 48 h, the medium was replaced with complete DMEM-5% FCS containing 5 μg/ml of the DNA polymerase-α antagonist aphidicolin ([69]; Sigma), and incubated 16 h prior release. The isoleucine/aphidicolin double block procedure was also used to highly synchronize NB324K human fibroblasts. G1/S, thymidine: a double thymidine block [70] was used to synchronize mouse fibroblasts. Cells seeded (2.5x10^5^ cells/P60 mm dish) in DMEM-5%FCS for 24h were incubated for 16h in fresh medium supplemented with 2 mM thymidine, a nucleoside that competes for thymine incorporation into DNA. The medium was removed and replaced by normal medium without thymidine for 9h, followed by a second 16h treatment in fresh medium with 2 mM thymidine. Cells were released from the G1/S blocks by removing the medium with the inhibitor, washing with PBS several times and adding fresh pre-warmed DMEM-5% FCS.

### Flow cytometric analysis

For viral antigens determination, cells were fixed for 24h in 70% ethanol at 4°C, permeabilized (PBS, 0.1% Triton X-100) for 20 min, and blocked (PBS, 0.1% Triton X-100, 1% FCS) for 10 min. Primary and secondary antibodies (mentioned below) were sequentially incubated for 1 h at 37°C in blocking solution. For DNA content determination, fixed and permeabilized cells were stained for 1 h in PBS, 0.1% Nonidet P-40, and 3 μM DAPI. All samples were analyzed in a BD Biosciences FACSCanto II Flow Cytometer equipped with 405, 488 and 633 nm laser lines. The BD FACSDiva (v6.1.2, BD Biosciences) and FlowJo (v9.3 for mac and v7.5 for PC, TreeStar) softwares were respectively used for sample acquisition and data analysis. The Cell Cycle Platform of FlowJo (v7.5) with Watson Pragmatic algorithm were used for quantitative analyses.

### Virus and synchronous infections

The prototype strain of the parvovirus Minute Virus of Mice (MVMp; [22]), referred simply as MVM for this study, was grown in NB324K cells and titrated as plaque forming units (pfu) as previously described [71]. Virus stocks were prepared from infections at low multiplicity, purification by density gradients free of empty capsids, and stored at −70°C [43,72]. For synchronous infections, the purified virus was added at a multiplicity of infection (moi) of 10 pfu/cell, in the media and timings accordingly to the synchronization methods, as follows. G1 arrest at confluence: the virus was inoculated in DMEM-0.1% FCS and, upon 1h adsorption, the arrest was maintained in DMEM-5% FCS at least 14 h prior sub-culturing. G1/S, aphidicolin: in cultures para-synchronized by low serum (mouse fibroblasts only), or isoleucine deprivation (mouse and human fibroblasts, the virus was inoculated in normal or isoleucine-deprived DMEM, respectively. After 1h adsorption, the inoculum was removed and cells were further highly synchronized at G1/S by culturing for 16 h in 5 μg/ml aphidicolin in DMEM-5%FCS. G1/S, thymidine: when synchronizing by the double thymidine block (TT), the 1 h virus adsorption was performed in DMEM-0.1% FCS supplemented with 2mM thymidine in the middle of the second block (8 h post-second thymidine addition), and afterwards the thymidine treatment was maintained 7 h further in fresh medium. Time of virus cycle in the figures is referred to either hours post-inoculation (hpi), post-serum addition (hps), post-subculturing (hpc), post-aphidicolin release (hpa), post-thymidine blocks (hpTT), or general post-release of the inhibitor (hpr).

### Infectious plasmids

The respective infectious molecular clones of the prototype (MVMp; [73]) and the immunosuppressive (MVMi, [74]) viral strains have been previously described [65,75]. Our MVM-derived genomic clones allowing single expression of each of the structural proteins (VP1/ΔVP2 (or VP1-only), and ΔVP1/VP2 (or VP2-only) plasmids), were constructed for MVMp by cDNA cloning [72], and for MVMi by mutating the minor splicing donor site and the VP1 specific open reading frame [42]. Genomic plasmids were transformed in the *Escherichia coli* JC8111 strain that permits deletion-resistant propagation of plasmid clones bearing terminal parvovirus palindromes [76].

### Stable expressions of MVM capsid proteins

Fibroblasts permanently expressing the capsid proteins (VPs) of MVMp were derived from the A9 and NB324K cell lines. Clones were obtained co-transfecting (at molar ratio 1:5) the selection plasmid pSV2neo (carrying the bacterial aminoglycoside phosphotransferase gene that confers resistance to neomycin analogous) with the pSVtk-VPs plasmid, which contains the coding regions of the VP1 and VP2 proteins of MVMp cloned under the control of the SV40-thymidine kinase enhancer-promoter [77]. Mouse cells were transfected by a conventional calcium phosphate precipitate method, whereas the human cells were electroporated as described [36,72]. Cells were maintained at low density in medium containing the selective antimetabolite geneticin (G418, Gibco) at 400 μg/ml active product for 2–4 weeks to allow the growth of resistant clones. Twenty pick-isolated clones were grown from each transfected cell line and screened for the expression of both VP1 and VP2 structural proteins by indirect IF and western-blot with the α-VPs serum (see below). Homogenates from several VPs-expressing clones allowed the purification of MVM empty capsids, as described for other parvovirus VP-expressing systems (e.g. [78–82], which showed hemagglutinating activity with mouse erythrocytes [71]. Eight transfected clones were selected and pooled from each cell type on the basis of their normal growth and morphology, viability in colony forming units assay [71], and VPs expression to levels fairly detectable by blot at the VP1:VP2 ratio of 1:5 found in the natural MVM infection [79,83]. The mouse and human pools of clones (respectively named MF-VPs and HF-VPs) showed stable VPs expression over the passages.

### Analysis of VPs expression

To determine VPs synthesis by immunoprecipitation (IP), cells were ^35^S-Met/Cys pulse-labeled for 20 min with 100 μCi/ml of (35S) methionine-cysteine (Pro-mixTM, Amersham) in methionine-free DMEM-10% dialyzed FCS supplemented with 5% normal medium. Cells were washed twice with cold PBS, scraped in 150 mM NaCl, 50 mM Tris pH 8.0, 1% NP40, 0.3% SDS, 0.5% β-mercaptoethanol, and incubated overnight at 4 oC with a 1/100 dilution of the α-VPs serum. Immune complexes were precipitated with protein A-Sepharose (10% wt/vol), washed with cold PBS, 0.05% NP40, and 1% BSA, and bound proteins subjected to 10% SDS-PAGE. Gels were fixed, dried and exposed for autoradiography to Kodak X- films. To determine VPs accumulation by western-blot, proteins resolved in polyacrilamide gels (10%SDS-PAGE) were transferred onto nitrocellulose membranes (Scheider & Schull) by a wet transfer (Trans blot Electrophoretic-transfer Cell, Biorad) for 1 h at 100 V, and developed with the α-VPs serum by a chemiluminescence method (ECL, Amersham).

### 2D phosphopeptides resolution

Cells were labeled with 2 mCi/ml of ^32^P-orthophosphate in phosphate-free medium as described [43], and the VPs proteins were isolated from cellular extracts by IP with the α-VPs serum, resolved by 8%SDS-PAGE, and blotted to membranes. The purified 32P-labelled VP2 protein was cut-off from the membranes and subjected to 2-D tryptic phosphopeptide analysis by digestion with N-tosyl-L-phenylalanine chloromethyl ketone (TPCK)-trypsin (sequencing grade, Boehringer) and resolution in 20x20 cm TLC plates (Merck, Darmstadt, Germany) following our reported methods [38,43]. Plates were exposed to a radioanalytic imaging system (Fujix BAS 1000, Fuji) for ten days.

### Cross-linking of capsid proteins

Infected or transfected cells washed in PBS were centrifuged and resuspended in 50 mM Hepes pH 8.0, 150 mM NaCl, 2 mM MgCl_2_, 1 mM EDTA (HNEM buffer), and proteases (0.1 mM phenylmethylsulfonyl fluoride (PMSF), 10 μg/ml leupeptin, 10 μg/ml pepstatin, 10 μg/ml aprotinin, 100 μg/ml TPCK) and phosphatases (20 mM glicerol phosphate, 5 mM NaF) inhibitors. Cells were lysed by ultrasounds at 4°C and the homogenates were clarified by centrifugation at 10000 g, 30 min at 4°C. Wherever indicated purified capsids were used as controls. The dimethyl suberimidate imidoester (DMS; Sigma) cross-linker, freshly dissolved in 50 mM in Hepes-NaOH pH 8.0, was added at 10 mM to the prewarmed samples, and cross-linking allowed to proceed for 10 min at 20°C as reported earlier [37]. The reactions were finished by adding ethanolamine to a final concentration of 0.36 M.

### Antibodies

To cellular proteins we used: a rabbit polyclonal (α-Raf, Santa Cruz Biotechnology) recognizing the conserved C-terminus of human Raf-1; a mouse monoclonal antibody (α-NPC; mAb414, BAbCO, Richmond, CA) as marker of the nuclear envelope; and the α-PCNA and α-BrdU antibodies mentioned below. Two anti-MVM antibodies were used in this study: for the general recognition of both capsid proteins independently of the configuration of structured epitopes, an antiserum (α-VPs) was raised in rabbit against denatured VP2, which coding region is common to VP1 [42,79,83]. For this purpose, purified virus like particles (VLP) self-assembled by VP2 expression in a baculovirus system [81] were subjected to preparative 8%SDS-PAGE. A small vertical strip was stained, the region of the gel corresponding to the VP2 protein was excised, and the protein was electro-eluted into chambers running at 24 V overnight in 100 mM glycine-20 mM Tris base-0.01% SDS as previously described [84]. The purified denatured VP2 was directly used to raise an antibody in rabbit by injection (100 μg per dose) a first dose emulsified in Freund´s complete adjuvant followed by three boost injections in incomplete adjuvant. Bleeding was done ten days after the last injection. This rabbit α-VPs antibody was probed to recognized efficiently VP1 and VP2 when denatured in western-blots [72], whereas failed to react with purified MVM capsids by immune-precipitation or electron immune-gold staining. Intact capsids and virions were recognized by a mouse anti-MVM monoclonal antibody (α-Mab, B7), which failed to react with isolated VP subunits or trimers [37,38], in agreement with the available genetic [39] and 3-D structural data on its specific recognition of a capsid epitope [40]. The NS1 non-structural protein was recognized by a polyclonal antibody raised in rabbit against a recombinant β-gal-NS1 fusion protein expressed in bacteria [85].

### Immunofluorescence

For subcellular localization of proteins by double-label indirect immunofluorescence (IF), cells seeded onto glass coverslips in dishes (Nunc) were washed twice with PBS and fixed for 8 min in 4% paraformaldehyde (PFA) at room temperature, or in Methanol:Acetone 1:1 at -20°C. Cells were permeabilized with 0.1% Triton X-100 in PBS and, after blocking in PBS, 0.1% Triton X-100, 1% FCS, the primary antibodies were applied diluted 1:50 to 1:500 in blocking buffer for 1 h at 37 oC. The bound IgG were visualized with a secondary goat anti-rabbit antibody conjugated to Texas red (TXRD) and with a goat anti-mouse antibody conjugated to fluorescein isothiocyanate (FITC) (Jackson Immuno Research Laboratories, Inc.; used at 1/500). The simultaneous antibody staining and FISH- hybridization for the analysis of MVM replication and gene expression was performed as reported elsewhere (Gil-Ranedo et al., *in preparation*). For *in situ* monitoring cellular DNA replication, cells cultured on coverslips were incubated with 20 μg/ml bromodeoxiuridine (BrdU, Sigma) for a 15 min- pulse, washed in PBS, and immediately fixed in methanol for 20 min at 4°C. The DNA was partially denaturized in 2N HCl for 1h at 37°C, and then neutralized washing three times in borate buffer (pH 8.5) and PBS. Samples were analyzed by IF with an anti-BrdU specific antibody (Boehringer). The Proliferating Cell Nuclear Antigen (PCNA), a cofactor of the DNA polymerase δ [86,87], was used as marker of S phase (PC10 antibody, Santa Cruz biotechnology). Samples were mounted in DAPI Fluoromont-G (Southern Biotech) and left to dry overnight. Phenotypes were scored by epifluorescence using a Zeiss Axiovert2000 inverted microscope coupled to a SPOT RT Slider digital camera and MetaVue 5.07 software, and images were taken in a Zeiss LSM 710 Laser Scanning confocal microscope and ZEN 2008 software. The images captured by 1.5 μm optical sections were processed using Fiji package. For each antigen, the scored cells were classified in three categories: mainly nuclear (N>C), comparable nuclear and cytoplasmic (N = C), and mainly cytoplasmic (N<C). The percentages of N/C subcellular distribution shown in the figures were obtained from at least 400 IF-stained cells accumulated from three to four consistent and independent experiments.

### VP2 and Raf-1 expression by baculoviruses

The recombinant baculoviruses of the *Autographa californica* (AcMNPV) species, expressing the wt VP2 of MVMp [81], or the Raf-22W constitutively active mutant form of the Raf-1 kinase [88], were grown in Sf9 cells and titrated as fluorescence focus (IFU). For expression of the recombinant proteins, H5 insect cells cultured at 27°C in TC100 medium supplemented with 5% FCS were infected at a multiplicity of 10 IFU/cell for each baculovirus and fixed in 4% PFA at 48–72 hpi following previously described methods [38].

### Nuclear transport of purified VP2 trimer in permeabilized cells

A previously described methodology [38] was followed with minor modifications. In brief, trimers of the VP2 protein (VP2t) were purified by continuous sucrose gradients from NB324K cells transfected with the pVP2-K153A plasmid at large scale as recently optimized [72]. A total amount of 200 ng of VP2t dialyzed against transport buffer (2 mM Mg-acetate, 20 mM Hepes pH 7.3, 110 mM K-acetate, 1 mM EDTA, 5 mM Na-acetate, 2 mM DTT) was used as substrate per reaction. HeLa cells permeabilized with 40 μg/ml Digitonin (Sigma) in DMEM for 5 min at 37°C were subjected to the transport reaction for 15 min at 37°C including 12 mg/ml rabbit reticulocyte lysate (RRL; Promega), protease inhibitors and energy sources (1 mM ATP, 5 mM creatine phosphate (Sigma), and 20 U/ml of creatine phosphokinase (Calbiochem). In some experiments the RRL was substituted by the purified α2 (0.15 μM) and β1 (0.15 μM) importins (Calbiochem), and Ran-GDP (2 μM). Transport controls were BSA conjugated with either the SV-40TAg NLS (PKKKRKVED) of the α2/β1 importin route, or the M9 domain (YNNQSSNFGPMK) of the transportin route. Peptides were conjugated to BSA coupled with fluor agents (Alexa Fluor 488, 594, and 647; Molecular Probes) by the chemical cross-linker SMCC (Calbiochem), cleaned by chromatography in PD10 columns (Amersham), and concentrated by centrifugation through filters (3 kDa Microcon, Millipore). For control inhibition of the nuclear import, an excess of free NLS peptide (3.5 μM), or 100 μg/ml of wheat germ agglutinin (WGA, Calbiochem), were added. The transport reaction was terminated by fixation in 4% PFA.

### Ethics statement

Animal experimental protocols were approved by the Ethical Committees of the Centro de Biología Molecular Severo Ochoa (CSIC-UAM) and Universidad Autónoma de Madrid for animal health research (permit number CEI 27–652) in strict accordance with Spanish national Royal Decree (RD 1201/2005) and international EU guidelines 2010/63/UE about protection of animals used for experimentation and other scientific purposes, and Spanish national law 32/2007 about animal welfare in their exploitation, transport and sacrifice.

## Supporting Information

S1 Fig Cell cycle progression and MVM induced arrest in synchronous mammalian fibroblasts. Bars illustrate the percentage of mouse (A9) and human (NB324K) fibroblasts at each of the cell cycle phases upon release from synchronization at (**A**) G1/S by isoleucine/aphidicolin (hpa), (**B**) G1 by growth to confluence (hpc), and (**C**) G1/S by a double thymidine block (hpTT). Values were obtained from the cell cycle plots shown in Figs 3–5. Cells were fixed by ethanol at the indicated hours post-release of the arrests, stained by DAPI, and their DNA content quantitated by flow cytometry. Note the abrupt cell cycle arrest at late S provoked by MVM infection in both fibroblast cell lines, starting since 10 hpa, 14 hpc, and 6 hpTT.
(TIF)

S2 Fig Distinct subcellular capsid assembly in infected mouse and human fibroblasts synchronized by DNA synthesis arrest. The figure illustrates at high magnification the precise subcellular distribution of assembled capsids in infected mouse (MFs) and human (HFs) fibroblasts at 9h post-release of a double thymidine block (9 hpTT). Capsid signals in the HFs panels have been digitally overexposed to allow a sharp visualization.
(TIF)

S3 Fig Inhibition of the nuclear translocation of MVM capsid subunits by density arrest signals in synchronously infected mammalian fibroblasts. **A**. Cytoplasmic capsid assembly in MFs. *Upper*: scheme of the experimental setting. *Lower*: confocal analysis of subcellular localization of viral antigens (VPs, Capsid, NS1) and genome replication (vDNA) at the indicated hours post-aphidicolin release without (hpa), or upon the previous addition of a saturating number of cells at 6 hpa (panels 18 hpa/12 h cells and 14 hpa/8 h cells). **B**. MVM capsid assembly defect in HFs. *Upper*: scheme of the experimental setting. *Lower*: Confocal analysis of subcellular distribution of the viral antigens and genome replication in synchronously infected HFs at 24 hpc either without, or subjected to density-arrest signals at 7 hours post-subculture (24 hpc/17 cells). α-MVM, addition of an excess of neutralization units of an MVM-antibody [39]. Scale bars 50 μm.
(TIF)

S4 Fig Analysis of VPs oligomerization in synchronous transfected human fibroblasts. HF-VPs syncronized by growth to confluence were subcultured at low density and cellular extracts prepared at the indicated hpc. Shown are protein samples subjected to chemical cross-linking by dimethyl suberimidate imidoester (DMS), resolved by 5%SDS-PAGE, and analyzed by western-blot with the α-VPs antibody. Arrows indicate the two types of VPs trimers.
(TIF)
