# Supplementary figures and images for: The Mammalian Cell Cycle Regulates Parvovirus Nuclear Capsid Assembly
Source: PLoS Pathog. 2015 Jun 11;11(6):e1004920. doi: 10.1371/journal.ppat.1004920 (PMC4466232; doi:10.1371/journal.ppat.1004920)

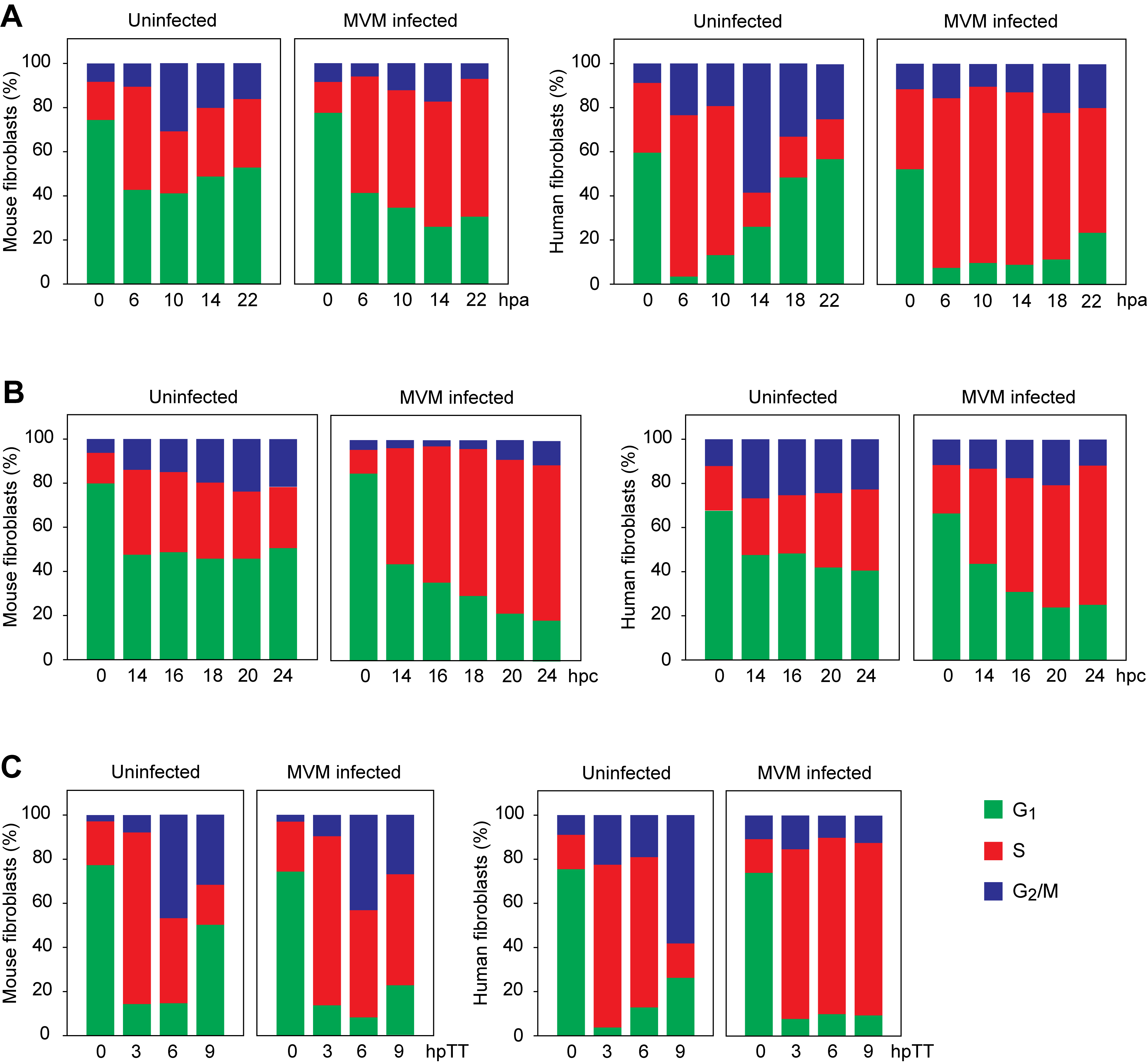

Supplement: S1 Fig — Bars illustrate the percentage of mouse (A9) and human (NB324K) fibroblasts at each of the cell cycle phases upon release from synchronization at (A) G1/S by isoleucine/aphidicolin (hpa), (B) G1 by growth to confluence (hpc), and (C) G1/S by a double thymidine block (hpTT). Values were obtained from the cell cycle plots shown in Figs 3–5. Cells were fixed by ethanol at the indicated hours post-release of the arrests, stained by DAPI, and their DNA content quantitated by flow cytometry. Note the abrupt cell cycle arrest at late S provoked by MVM infection in both fibroblast cell lines, starting since 10 hpa, 14 hpc, and 6 hpTT. (TIF) [file ppat.1004920.s001.tif]

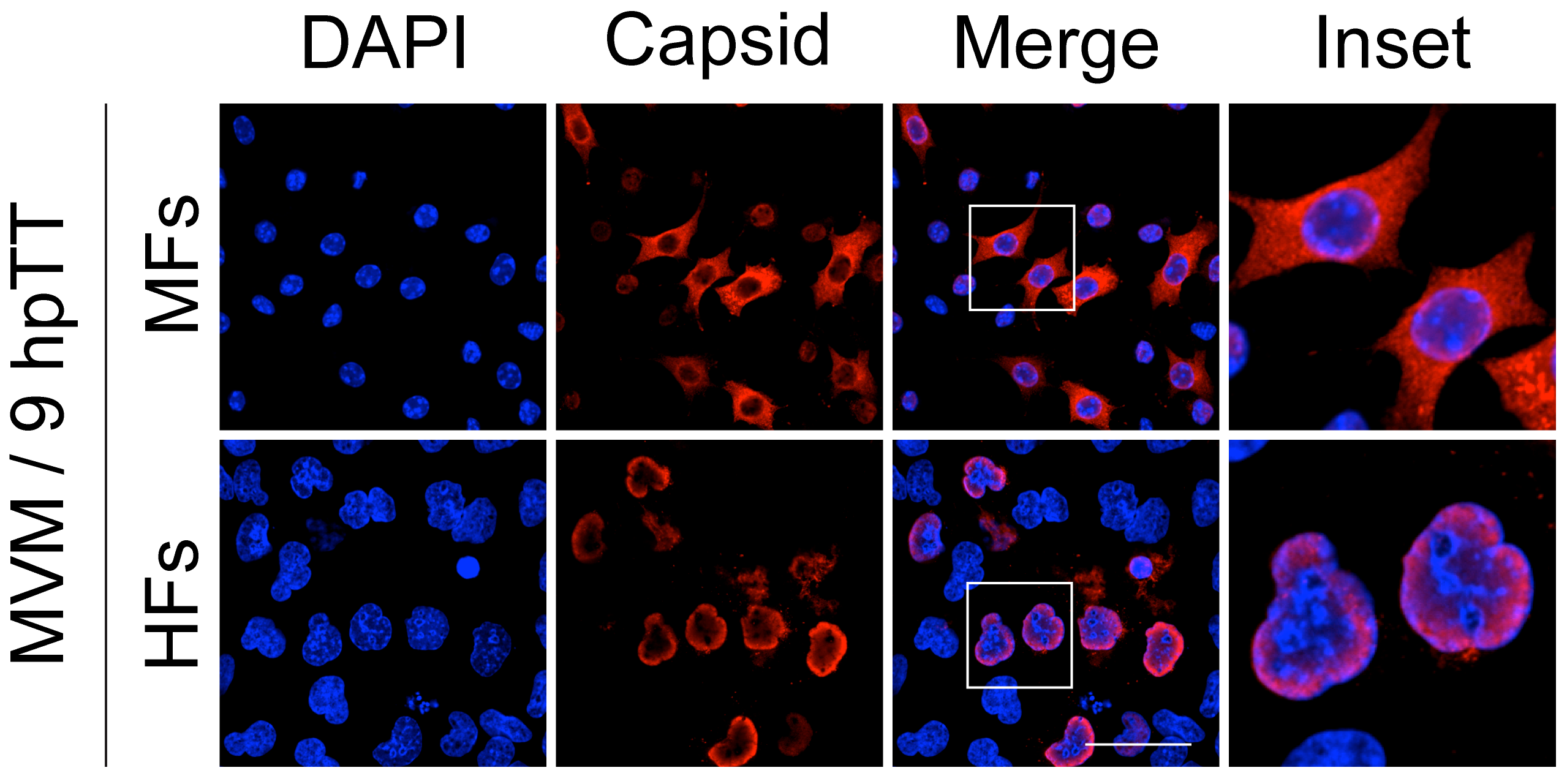

Supplement: S2 Fig — The figure illustrates at high magnification the precise subcellular distribution of assembled capsids in infected mouse (MFs) and human (HFs) fibroblasts at 9h post-release of a double thymidine block (9 hpTT). Capsid signals in the HFs panels have been digitally overexposed to allow a sharp visualization. (TIF) [file ppat.1004920.s002.tif]

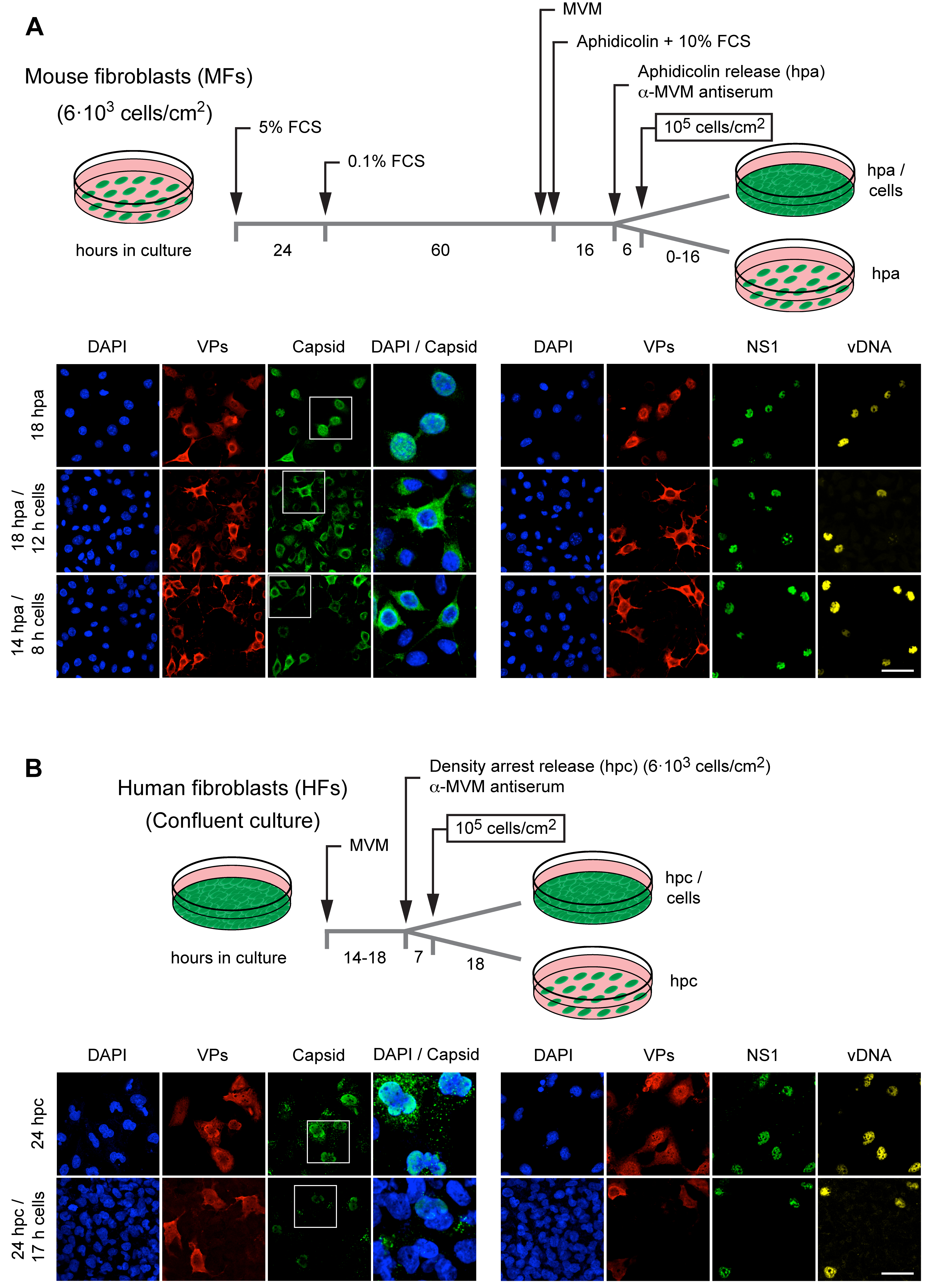

Supplement: S3 Fig — A. Cytoplasmic capsid assembly in MFs. Upper: scheme of the experimental setting. Lower: confocal analysis of subcellular localization of viral antigens (VPs, Capsid, NS1) and genome replication (vDNA) at the indicated hours post-aphidicolin release without (hpa), or upon the previous addition of a saturating number of cells at 6 hpa (panels 18 hpa/12 h cells and 14 hpa/8 h cells). B. MVM capsid assembly defect in HFs. Upper: scheme of the experimental setting. Lower: Confocal analysis of subcellular distribution of the viral antigens and genome replication in synchronously infected HFs at 24 hpc either without, or subjected to density-arrest signals at 7 hours post-subculture (24 hpc/17 cells). α-MVM, addition of an excess of neutralization units of an MVM-antibody [39]. Scale bars 50 μm. (TIF) [file ppat.1004920.s003.tif]

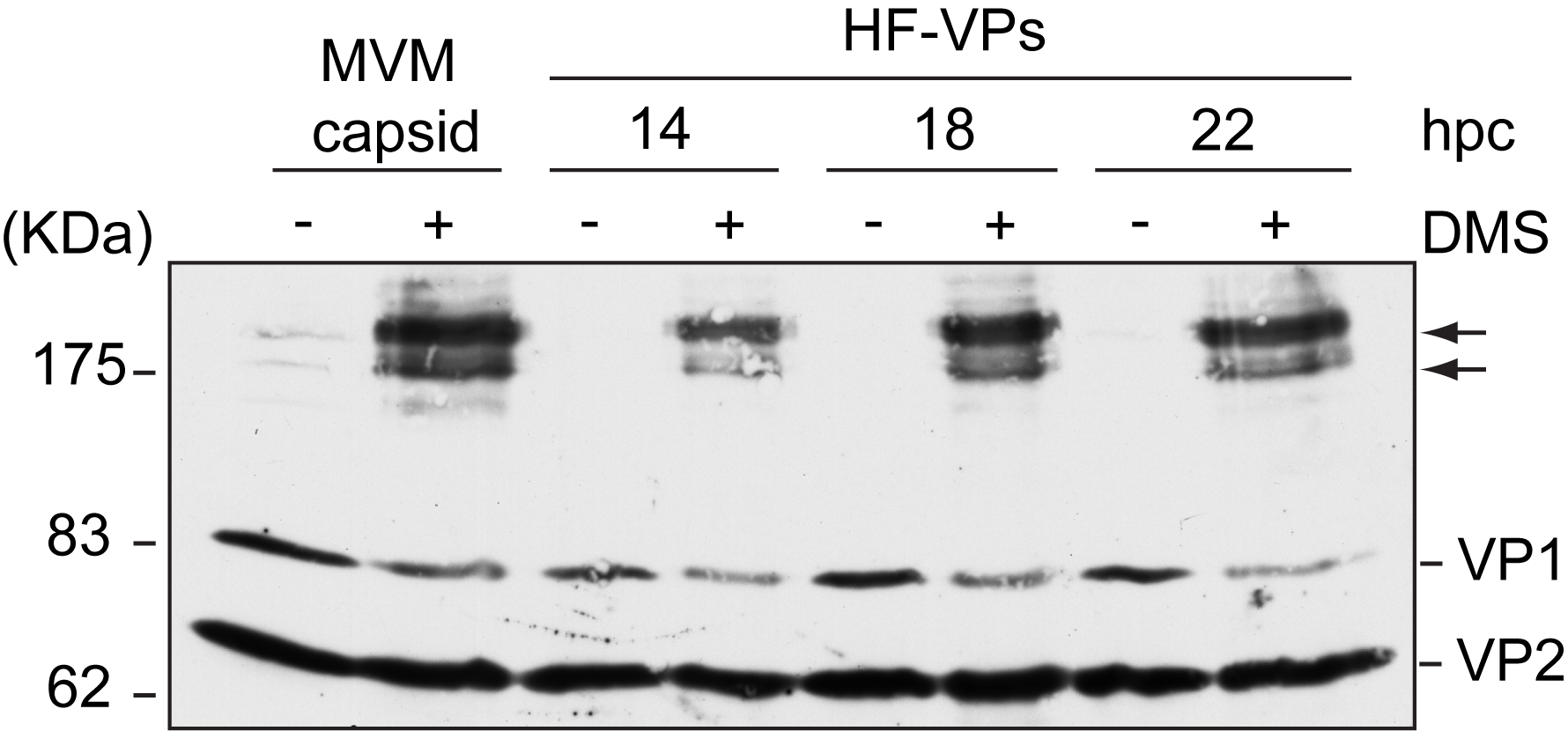

Supplement: S4 Fig — HF-VPs syncronized by growth to confluence were subcultured at low density and cellular extracts prepared at the indicated hpc. Shown are protein samples subjected to chemical cross-linking by dimethyl suberimidate imidoester (DMS), resolved by 5%SDS-PAGE, and analyzed by western-blot with the α-VPs antibody. Arrows indicate the two types of VPs trimers. (TIF) [file ppat.1004920.s004.tif]
